# Cas9-AAV6-engineered human mesenchymal stromal cells improved cutaneous wound healing in diabetic mice

**DOI:** 10.1038/s41467-020-16065-3

**Published:** 2020-05-18

**Authors:** Waracharee Srifa, Nina Kosaric, Alvaro Amorin, Othmane Jadi, Yujin Park, Sruthi Mantri, Joab Camarena, Geoffrey C. Gurtner, Matthew Porteus

**Affiliations:** 1 Department of Pediatrics, Stanford University School of Medicine, Stanford, CA 94305 USA; 2 Program in Stem Cell Biology and Regenerative Medicine, Stanford University School of Medicine, Stanford, CA 94305 USA; 3 Department of Surgery, Stanford University School of Medicine, Stanford, CA 94305 USA

**Keywords:** CRISPR-Cas9 genome editing, Stem cells, Mesenchymal stem cells

## Abstract

Human mesenchymal stromal cells (hMSCs) are a promising source for engineered cell-based therapies in which genetic engineering could enhance therapeutic efficacy and install novel cellular functions. Here, we describe an optimized Cas9-AAV6-based genome editing tool platform for site-specific mutagenesis and integration of up to more than 3 kilobases of exogenous DNA in the genome of hMSCs derived from the bone marrow, adipose tissue, and umbilical cord blood without altering their ex vivo characteristics. We generate safe harbor-integrated lines of engineered hMSCs and show that engineered luciferase-expressing hMSCs are transiently active in vivo in wound beds of *db*/*db* mice. Moreover, we generate PDGF-BB- and VEGFA-hypersecreting hMSC lines as short-term, local wound healing agents with superior therapeutic efficacy over wildtype hMSCs in the diabetic mouse model without replacing resident cells long-term. This study establishes a precise genetic engineering platform for genetic studies of hMSCs and development of engineered hMSC-based therapies.

## Abstract (web summary)

Human mesenchymal stromal cells are a promising source for cell-based therapies. Here the authors use Cas9 to engineer lines that secrete PDGF-BB and VEFGA for improving wound healing.

## Introduction

Genetic engineering of mesenchymal stromal cells can strengthen their therapeutic potentials and fill the knowledge gap of their biological properties. Recently, approved human MSC (hMSC) products and many investigational studies employ autologous and allogeneic hMSCs as treatment for inflammatory diseases and tissue injury^1–6^. The lack of long-term MSC engraftment in recipient tissue post transplantation led many to speculate that they confer immunomodulatory benefits and facilitate tissue repair through a ‘hit-and-run’ signaling mechanism without replacing the injured tissue^7–9^. The ability to culture MSCs long-term is beneficial for meeting large clinical dosing demands^10^, while their short-term presence following delivery can be advantageous for transient intervention in injured tissue and disease microenvironments. In addition, low risk for adverse events and ectopic tissue formation following systemic and local tissue delivery of cultured hMSCs in various clinical trials strongly indicates clinical safety of MSC-based therapy^4,11^. Despite promising therapeutic benefits, major criticisms of MSC-based therapy are rooted in the lack of knowledge of their mechanisms of action and inconsistencies in clinical outcomes^2,12^. In addition to genetically studying MSCs, ex vivo genetic engineering approaches can provide an opportunity to engineer MSC-based therapeutics with known mechanism of action, while capitalizing on large scale production and the established safety profile of MSCs to benefit patients.

However, the ability to genetically manipulate or engineer hMSCs for preclinical and clinical studies remains limited. While extra-chromosomal manipulation such as transient transgene and regulator RNA expression via plasmid/mRNA transfection and non-integrating viral vectors, or random transgene integration into the genome by integrating viral vectors have been pioneered in hMSCs, the methods collectively lack the ability to make changes to the genome in site-specific manners. Precise genome editing, on the other hand, allows functional manipulation by gene disruption, DNA sequence editing, and transgene integration at desired loci of the genome. In clinical settings, while transient gene expression or random gene integration might be adequate considering short term presence of MSCs in vivo, it might not be highly effective due to disadvantages of the engineering methods. Non-integrating vector-based engineering can lead to low episomal expression from viral vectors, degradation, or loss of non-integrating transgene expressing RNA/DNA elements with cell division^13–16^. Semi-random gene integration by retroviral and lentiviral vectors, though capable of stable expression that is preserved through cell division, constrains the manipulation only to the random integrase-targeted sites that cause heterogeneity in gene expression level and can also lead to oncogenic mutations or essential gene disruption^17–20^. Nuclease-based site-specific genome-editing techniques, such as engineered zinc finger nuclease- (ZFN-) assisted technology pioneered in hMSCs by Benabdallah et al.^21^, can provide safer, specific, and stable manipulation of the MSC genome and its expression. However, designing protein-based DNA recognition domains for ZFNs or TALENs is labor intensive. Therefore, we aimed to utilize CRISPR-based technology using Cas9 nuclease, whose target specificity is directed by easily designed single-guide RNAs (sgRNAs), for site-specific genome editing in hMSCs. Our recently developed Cas9-adeno-associated virus serotype 6 (AAV6)-mediated genome-editing tools, which can perform high efficiency, site-specific genome editing in hematopoietic stem and progenitor cells, provides a foundation for this study^22,23^.

An area in which engineered MSCs can be used therapeutically is impaired wound healing. Suboptimal physical environment and dysregulated biochemical crosstalk between immune cells, fibroblasts, keratinocytes, and endothelial cells result in prolonged inflammation and repressed new skin formation in chronic wound healing^24^, which requires clinical interventions in addition to standard wound care. MSC-based therapy has a potential to address current limitations of wound therapy and can be further engineered to enhance therapeutic effects^5,6^. Although multiple growth factors and cytokines are required to regulate wound healing^25^, only recombinant platelet-derived growth factor beta (PDGF-BB) topical gel, Regranex®, has been FDA-approved for treatment of diabetic lower extremity ulcers with up to 50% success^26^. Such treatment shows no efficacy in pressure ulcers or venous stasis ulcers, suggesting that PDGF-BB alone may not be sufficient for the general population affected by chronic wounds. Although MSCs are known to secrete multiple trophic factors that promote regeneration, no advanced trials or FDA-approved products exist for wound therapy, and effective MSC-based treatments of wounds needs improvements. In addition to the lack of multi-factor treatment, maintaining high tissue availability of topically applied recombinant growth factors is difficult in the proteolytic wound environment^25^.

Here, we tailor the Cas9-AAV6 genome-editing platform to ex vivo primary human MSCs, demonstrate system compatibility across tissue sources for site-specific safe harbor gene integration, and characterize therapeutic efficacy of Cas9-AAV6-engineered hMSCs as putative treatment of impaired wound healing. We demonstrate that the Cas9-AAV6 tool is compatible with hMSC genome editing and the resulting engineered hMSCs can act as a transient therapeutic agent in the wound bed of *db*/*db* mice and as source of high concentration of therapeutic factors to locally treat impaired cutaneous wound healing in the diabetic mouse model.

## Results

### Cas9-AAV6-targeted gene integration is effective in hMSCs

Successful implementation of the Cas9-AAV6 tool relies on effective delivery of its components. As previously established in human hematopoietic stem and progenitor cells^22,23^, we aimed to deliver nucleases via electroporation and homologous repair templates via AAV6 transduction to achieve transgene integration in hMSCs. First, we optimized conditions for electroporation of RNA into hMSCs. We found that both the pulsing protocol used and the buffer that the cells were suspended in can determine transfection efficiency and viability of hMSCs, and identified that suspension of cells and RNA in Opti-MEM® and pulsing with program CM-119 on the Lonza 4D Electroporator led to the most effective delivery and achieved high efficiency electroporation of reporter eGFP mRNA with more than 90% recovery of viable GFP-expressing (GFP^+^) human bone marrow-derived (hBM-) MSCs at 24 hours post electroporation (Supplementary Fig. 1a). Although the resulting GFP^+^ population retained GFP expression for at least 7 days, we observed exponentially declining fluorescence intensity with a half-life of ~1 day (Supplementary Fig. 1b). This suggests rapid degradation of mRNAs and demonstrates that MSCs modified with mRNA would have only have transient engineered activity with rapidly declining potency. Next, we utilized recombinant AAV6 vectors containing GFP expression cassettes to measure successful transduction in hBM-MSCs. Although AAV6 does not have known tropism for hMSCs, we successfully achieved transient expression of the fluorescence protein with the help of electroporation prior to AAV6 incubation in up to 65% of treated hBM-MSCs (Supplementary Fig. 1c). We further determined that this electroporation-aided transduction (EAT) was optimal at incubation ratio of 1 × 10^5^–2 × 10^5^ vector genomes per cell (Supplementary Fig. 1d). Having optimized these delivery methods, we proceeded to demonstrate effectiveness of the Cas9-AAV6 genome editing in hMSCs.

**Fig. 1:**
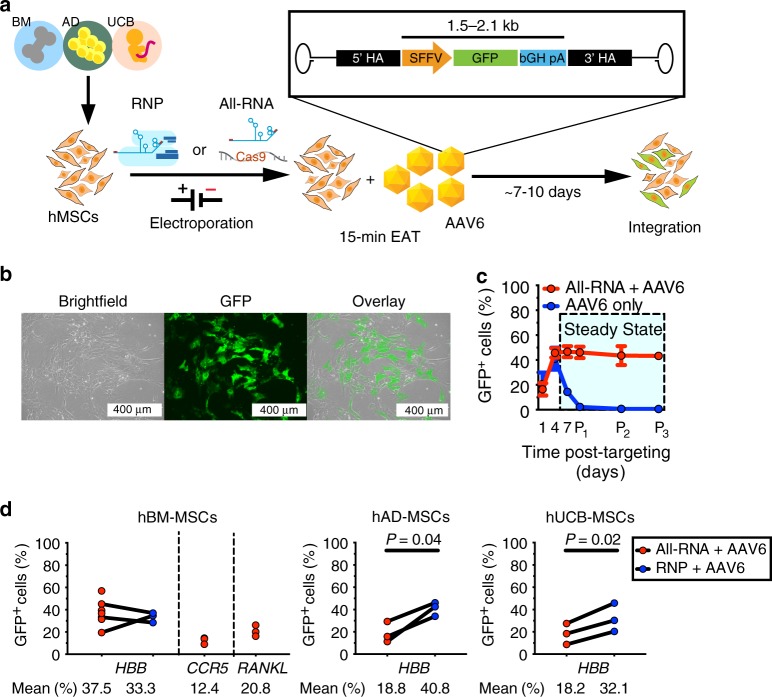
The Cas9-AAV6 platform is an effective and versatile tool for genome editing in human MSCs. **a** Schematics outline procedures for genome editing using the Cas9-AAV6 platform in human MSCs derived from the bone marrow (BM), adipose tissue (AD), and umbilical cord blood (UCB) for site-specific transgene integration. MS-modified guide RNAs and Cas9 nuclease are electroporated as All-RNA cocktail or RNP complex. Then, homologous repair templates containing GFP reporter cassette insert are delivered by electroporation-aided transduction (EAT) of AAV6 vectors for 15 min during recovery period. Integration of GFP-overexpression cassette can be detected ~7–10 days later. HA: homology arm **b** Representative single-channel and overlay images of a GFP^+^ hBM-MSC colony originated from low-density seeding (220 cells/cm^2^) one passage in culture after nuclease electroporation and EAT. Scale bars represent a distance of 400 µm. GFP^+^ colony formation is observed in targeting from all 12 biological hMSC donors described in this study. **c** Dots and lines represent average frequencies of GFP^+^ cells in hBM-MSCs (biological replicates: *n* = 6) at different timepoints after *HBB* locus targeting using All-RNA nuclease and AAV6 EAT or AAV EAT only. P_i_ signifies the end of the i^th^ passage post targeting. Error bars represent standard error of mean. **d** GFP^+^ cell frequencies at one passage post targeting of hBM-MSCs (biological replicates: *n* = 7), hAD-MSCs (*n* = 3), and hUCB-MSCs (*n* = 3), which were targeted with All-RNA cocktails or RNP complexes followed by AAV6 EAT are represented in dot plots for indicated gene loci (*HBB*, *CCR5*, and *RANKL*). Connected dots represent hMSCs from the same human donors. Mean indel frequencies were compared between two modes of targeting and two-tailed *p*-values from paired *t*-test are shown for significant differences of mean. Source data are available in the Source data file.

To demonstrate that the Cas9-AAV6 platform can precisely alter the genome of hMSCs, we utilized the system to integrate transgenes harboring GFP expression cassettes into the genomes of human bone marrow-derived (hBM-), adipose tissue-derived (hAD-), and umbilical cord blood-derived (hUCB-) MSCs (Fig. 1a). We first confirmed that Cas9 nuclease, together with chemically modified sgRNA, can highly efficiently generate double-stranded breaks (DSBs) and insertion-deletion mutations (indels) in hBM-MSCs, hAD-MSCs, and hUCB-MSCs in putative safe harbor loci for hMSCs (Supplementary Fig. 2, target sequences listed in Supplementary Table 1) (symbols: (−i) deletion of i nucleotides (nt) around cut site, (+i) insertion of i nt around cut site). Of note, *HBB*, *CCR5*, and *RANKL* are not expressed in MSCs and could be considered safe harbors for this cell type. Our results show that Cas9 is effective in hMSCs when electroporated as mRNAs in a mixture with sgRNAs (All-RNA) or as ribonucleoprotein (RNP) complexes with sgRNAs (Supplementary Fig. 2b). Although different modes of Cas9 delivery may be employed to generate indels with similar signatures (Supplementary Fig. 2c), we found that the off-target activity of the nuclease delivered as All-RNA mixture targeting the *HBB* locus is significantly higher than that of RNP, and that off-target specificity is further improved with the use of high-fidelity Cas9 (Supplementary Fig. 2e, f). Of note, high off-target indels observed as a result of targeting with this particular sgRNA sequence at *HBB* is expected as all three nucleotide mismatches at the off-target site reside in the distal-most positions from the PAM motif, which are known to be least significant for specificity^27^. Our results highlight the importance for selection of unique sgRNA with low off-target potentials and the use of RNP system or high-fidelity Cas9 to avoid off-target DSBs.

For each unique sgRNA sequence, we utilized an AAV6-packaged repair template containing a GFP expression cassette flanked by arms of homology centered around the break site as donor DNA molecule for DSB repair (Supplementary Table 1). Following Cas9-AAV6 delivery, GFP^+^ colonies of targeted hMSCs appeared in the culture of low-density seeded (~220 cells/cm^2^) hMSCs within one passage (Fig. 1b). We further determined timing of gene integration by quantifying the GFP^+^ fraction over a time course of three passages with flow cytometry. When targeted with sgRNA, Cas9, and AAV6 repair template, frequencies of GFP^+^ hBM-MSCs peaked at 4 days post targeting and subsequently reached steady state in as early as 4–7 days and sustained through at least three passages (Fig. 1c). Although AAV6 vector transduction alone also generated a GFP^+^ population, which can be observed 4 days later, the population effectively diminished after one passage in culture. This early emergence and eventual disappearance of GFP^+^ population in cells targeted with AAV6 without nucleases is consistent with the kinetics of gene expression from AAV episomes, which are diluted by cell proliferation. The transient expression of GFP with rapid decay kinetics following AAV only delivery demonstrates the weakness in AAV alone as an engineering method to enhance MSC potency. Our results show that Cas9-induced DSBs are necessary for high frequency of genome integration of the transgene carried by AAV6 vectors and that the integration occurred rapidly.

We performed gene integration in hMSCs from multiple human donor lines using sgRNA-AAV6 pairs to target the *HBB*, *CCR5*, and *RANKL* gene loci (Fig. 1d, Supplementary Table 1). At steady state, using the *HBB*-targeting sgRNA with two modes of Cas9 delivery (All-RNA vs RNP) in combination with AAV6 template transduction yielded comparable gene targeting frequencies in hBM-MSCs, with average targeting frequencies of 32.6 ± 12.9% and 33.3 ± 4.4%, respectively (gating strategies shown in Supplementary Fig. 3). In hBM-MSCs, we also achieved integration frequencies of 12.4 ± 3.0% when targeting at *CCR5* and 20.8 ± 5.0% at *RANKL*. Furthermore, we achieved gene targeting via *HBB* locus integration in hAD-MSCs (All-RNA: 18.8 ± 9.4%, RNP: 40.8 ± 6.2%) and hUCB-MSCs (All-RNA: 18.2 ± 9.4%, RNP: 32.1 ± 12.8%), with significantly higher frequencies with RNP-based targeting (*p*-values < 0.05, two-tailed paired *t*-test) (Fig. 1d). These results show that primary human MSCs from different tissue sources can be efficiently engineered using the Cas9-AAV6 system. The delivery of Cas9 can be done by either mRNA or by protein delivery with both giving high efficiencies. By using methods like nuclease electroporation and AAV vector transduction to transiently deliver the Cas9-AAV6-system, we can generate engineered hMSCs with lasting transgene expression in culture. We note that the efficiency of integration may vary based on the size of inserts (Supplementary Fig. 3d).

### Cas9-AAV6-targeted hMSCs retain ex vivo characteristics

**Fig. 2:**
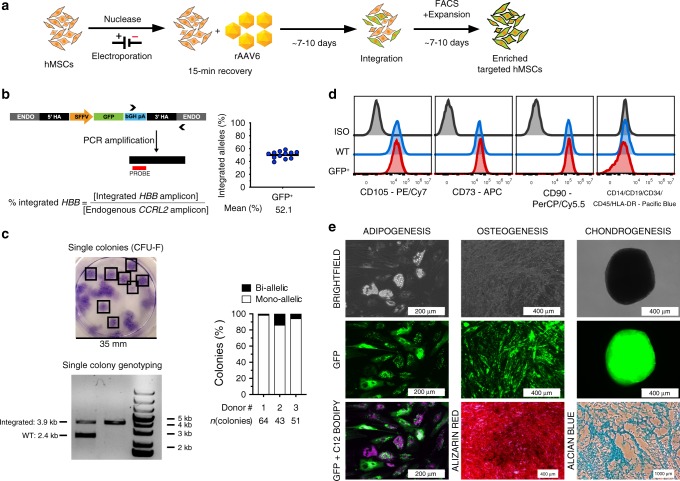
Genome-editing procedures and gene integration at *HBB* preserved ex vivo hMSC characteristics. **a** *HBB*-targeted GFP^+^ population of hMSCs were enriched by FACS for genotypic and phenotypic characterization. **b** Genomic DNA of the GFP^+^ population was subjected to digital droplet PCR-based quantification of transgene-integrated *HBB* alleles. Absolute quantification of integrated allele was normalized to that of endogenous *CCRL2* locus. Plot represents fraction of integrated alleles in sorted GFP^high^ hMSC population (biological replicates: *n* = 12) targeted with either All-RNA or RNP nucleases and AAV donors. **c** (Top left) Representative image shows crystal violet staining of colony-forming units (CFU-F). Boxes enclose single colonies. (Bottom left) Representative agarose gel image shows size-separation of integrated and non-integrated *HBB* amplicons. Genotyping is repeated in parallel PCR reactions for each colony derived from three different hMSC donors, which yielded similar amplicon distribution as a result of gel electrophoresis. (Right) Plot represents frequency of colonies with mono-allelic and bi-allelic integration genotypes in three different biological hMSC donors. Numbers of colonies screened for each donor is listed underneath the plot as *n*(colonies). **d** Staggered histograms show representative distribution of CD105, CD73, and CD90 expression level and the lack of hematopoietic markers expression on the surface of sorted GFP^+^ hMSCs compared to wild-type cells (WT) and isotype controls (ISO). **e** Representative image panel represents morphology (brightfield), GFP expression (green), and characteristic staining of differentiated sorted GFP+ hMSCs. Accumulated lipid droplets in adipogenic differentiation were stained with C12-BODIPY or LipidTOX® stains (magenta). Calcium deposition in osteogenic differentiation was confirmed with Alizarin Red staining. Chondrogenesis was confirmed with pellet formation and Alcian Blue staining of pellet sections. Distances are indicated by scale bars. Tri-lineage differentiation potential is confirmed in all hMSCs derived from 12 different donors. Source data are available in the Source data file.

To confirm that the Cas9-AAV6 machinery can manipulate the genome of hMSCs without causing unintended changes in cellular functions and phenotypes, we determined the effects of gene integration at *HBB* locus on MSC characteristics as defined by the 2006 criteria from the International Society for Cellular Therapy (ISCT)^28^. We purified GFP^+^ hMSCs by fluorescence-activated cell sorting (FACS) at one passage after the gene integration procedure (Fig. 2a) and assessed their long-term transgene expression, genotype, immunophenotypes, and tri-lineage differentiation potentials. Flow cytometry analysis revealed that GFP^+^ hBM-MSCs retained their transgene expression for three passages, maintaining more than 90% purity (Supplementary Fig. 3f). Of note, the robust GFP expression observed indicates that targeted transgene integration into the *HBB* locus permits long-term overexpression in hMSCs. Next, we confirmed genomic DNA integration of the GFP expression cassette at the *HBB* locus in purified GFP^+^ cells by detecting the junction between transgene and genomic sequence downstream of the 3′ homology arm using a digital droplet PCR assay. In pools of GFP^+^ hBM-MSCs, we measured concentrations of integrated *HBB* alleles relative to those of endogenous *CCRL2* alleles. We consistently observed relative integrated *HBB* amplicon concentration of ~50% across multiple biological samples, which suggested predominant mono-allelic over bi-allelic integrations (Fig. 2b). To further support this notion, we genotyped single colonies derived from low-density seeded culture of GFP^+^ hMSCs, using a PCR-based assay to differentiate non-integrated and integrated *HBB* alleles based on amplicon sizes. Consistent with results from ddPCR assay, the majority of the colonies are heterozygous for gene integration at the *HBB* locus (Fig. 2c).

To compare ex vivo characteristics of gene-integrated and wild-type (WT) hMSCs, we used flow cytometry and tri-lineage differentiation assays to compare surface marker expression and differentiation potentials between hMSCs from the same human donors. Notably, as *HBB* and their potential off-target sites have no role in MSC function, disruption at the gene locus should not directly interfere with MSC properties. We found that both WT and GFP^+^ hMSCs expressed the same levels of CD105, CD73, and CD90, and in the absence of CD14, CD19, CD34, CD45, and HLA-DR expression (Fig. 2d), which is consistent with guidelines set by the International Society for Cellular Therapy. In addition, GFP^+^ hMSCs and their WT counterparts accumulated lipid droplets as shown by incorporation of BODIPY^TM^-C12 or LipidTOX^TM^ dye after 14 days in adipogenic induction medium. Cells from the same populations deposited calcium, as indicated by Alizarin Red staining, when cultured in osteogenic medium. They also formed chondrogenic pellets whose proteoglycan could be stained by Alcian Blue. Moreover, differentiated targeted cells retained their GFP expression throughout the culturing periods in induction media, showing no evidence of integrated cassette-silencing during differentiation (Fig. 2e). With no changes in immunophenotypes and tri-lineage differentiation potentials following gene integration at *HBB*, we conclude that the Cas9-AAV6 platform can alter the genome of hMSCs without disrupting their ex vivo properties, and that the *HBB* locus can serve as a safe harbor for transgene integration in hMSCs that permits protein overexpression. The latter is a somewhat surprising result given that *HBB* is only expressed in erythroid cells and might be predicted to be non-permissive for expression in hMSCs. Moreover, because no GFP^+^ cells emerged from the non-GFP-expressing (GFP^−^) population after sorting, we conclude that all gene targeting tool components only functioned transiently during the first days post targeting. Lastly, as subsequently differentiated tri-lineage progenies of gene-integrated hMSCs retained their transgene expression, we conclude that the *HBB*-integrated transgenes can maintain function without gene silencing in differentiated hMSCs. On the basis of our results, hMSCs can tolerate both Cas9-induced DSBs and AAV6 vectors well, despite minimal toxicity (Supplementary Fig. 3) and potential transient transcriptional responses^29^. We note that the sgRNA targeting *HBB* has been extensively evaluated for off-target activities, which can be minimized with the use of high-fidelity (HiFi) Cas9^30^.

### Locally injected engineered hMSCs briefly survive in wounds

We further confirmed that engineered hMSCs can carry out their transgene functions in the recipient tissue in vivo. Survival and bioactivity of engineered hMSCs following injection can determine their usage and effectiveness as therapeutics. Using our Cas9-AAV6 platform, we integrated 3.2 kilobases (kb) of a bi-cistronic firefly luciferase (Fluc) and GFP overexpressing cassette into the *HBB* locus of hBM-MSCs to generate dual-reporter-expressing Fluc^+^GFP^+^ hBM-MSCs (Abbreviation: Fluc MSCs) for live animal bioluminescence imaging. Following genome editing, we purified GFP^+^ cells by FACS (Supplementary Fig. 4) and confirmed luciferase activity with in vitro d-luciferin substrate incubation.

**Fig. 3:**
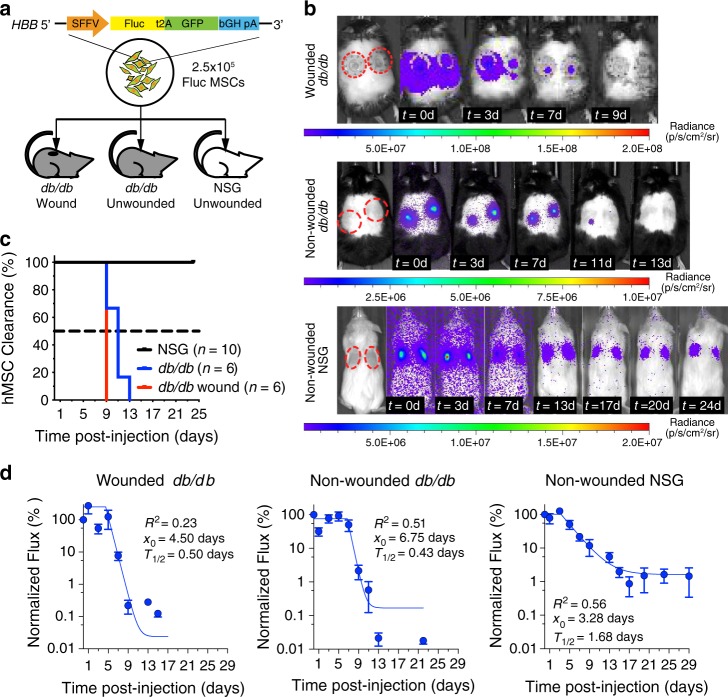
Both immunogenicity and poor survival contribute to clearing of subcutaneously injected hBM-MSCs in mouse xenotransplantation models. **a** Cas9-AAV6-engineered bi-cistronic firefly luciferase^+^GFP^+^ hBM-MSCs (Fluc MSCs; 2.5 × 10^5^ cells/injection) were injected into 6-mm cutaneous wound beds of *db*/*db* mice on the day of wounding (wounds: *n* = 6), and subcutaneously in unwounded *db*/*db* mice (injections: *n* = 6) or in NSG mice (*n* = 10). In vivo luciferase activities were measured over the course of up to 24 days with d-luciferin intraperitoneal injection and IVIS imaging systems. **b** Representative overlay bioluminescence and still animal images show source and intensities of emitted photons. Representative time series show decreasing luciferase activities of Fluc MSCs in injected mice overtime. **c** Kaplan–Meier’s plot shows time to complete clearance of luciferase activities from individual injection sites in each subject groups. **d** Dots and trendlines represent average net flux measured from each injection sites over time normalized to those measured on the day of Fluc MSC injection. Net flux values above baseline are shown. Dots and error bars represent mean value and standard deviation. Trendlines represent best plateau and one-phase decay curve fit of normalized flux. R2, *x*_0_, and *T*_1/2_ represent goodness of fit, plateau duration, and half-life, respectively. Complete clearance is determined by baseline photon flux measured from un-injected control animals. Luminescence flux from wounds or skin injected with Fluc MSCs was measured over time. Source data are available in the Source data file.

To establish therapeutic windows for engineered hMSCs in the leptin-deficient *db*/*db* mouse model of impaired wound healing, we used in vivo imaging to track bioluminescence activity of 2.5 × 10^5^ Fluc MSCs in cutaneous wound beds following wounding and a single local administration of engineered cells. Moreover, to examine whether the recipients’ immune system and MSC-intrinsic ability to survive, or the lack thereof, determine such therapeutic window, we performed analogous experiments in non-wounded skin of *db*/*db* mice, in which immune activity is at the baseline without wound-related inflammation, and in immunocompromised NSG mice where baseline immune activity is minimal (Fig. 3a). At the time of imaging, substrate d-luciferin solution was injected intraperitoneally into recipient mice and resulting photon generated as a result of luciferase enzyme activity was detected by optical imaging devices. Throughout the observation period of up to 24 days, we detected luciferase activities exclusively at the subcutaneous Fluc MSC injection sites in all three recipient groups (Fig. 3b). On the basis of such observations, we inferred that locally injected Fluc MSCs remained largely in the injection sites upon administration. In addition, we compared total duration of Fluc MSC survival in each recipient group by determining the time at which luciferase activities reached the baseline for each injection site. We found that total Fluc MSC survival time of up to 9 days in *db*/*db* wound beds are significantly shorter than those in non-wounded *db*/*db* skin of up to 13 days (*p*-value = 0.019, Log-rank test), and that total survival time in both *db*/*db* groups are significantly shorter than in non-wounded NSG skin (*p*-values ≤ 0.0001, Log-rank test), in which luciferase activities remained detectable at 24 days post injection (Fig. 3b, c). To determine kinetics of MSC survival and expression of the transgene, we measured luminescence flux in the wound areas or injected skin sites over time, and performed non-linear curve fit to measurements from each recipient group. Following a single injection on the day of wounding in *db*/*db* mice, luciferase activity remained relatively stable (*x*_0_) for ~4.5 days post injection before signals started to exponentially decline and reached baseline around 9 days post injection with a half-life (*T*_1/2_) of 0.5 days (Fig. 3d, left panel). Although luciferase activities declined in non-wounded *db*/*db* skin at a comparable rate (*T*_1/2_ = 0.43 days), they remained stable slightly longer in the initial plateau phase (*x*_0_ = 6.75 days) (Fig. 3d, middle panel). Lastly, in non-wounded NSG skin, activities stabilized in a plateau shortly (*x*_0_ = 3.28 days) but declined at a relatively slower rate (*T*_1/2_ = 1.68 days) and remained detectable with ~1% of original activity at the end of the 24-day observation period (Fig. 3d, right panel).

To determine whether engineered hMSCs can elicit adaptive immune responses in *db*/*db* mice, we compared total clearing time and kinetics of luciferase activity in the wounds of naive and pre-exposed *db*/*db* mice. Wounds of *db*/*db* mice either received no injection or an injection of 2.5 × 10^5^ Fluc MSCs on the day of wounding, then we injected wounds from both groups with the same dose of cells at 14 days post wounding and compared luciferase activities following day 14 injection. As a result, we observed significantly different total survival duration (*p*-value = 0.0044, Log-rank test), and that, while half-life of decline are comparable in naive and pre-exposed mice, we only observed the initial activity plateau in naive mice, but not in the pre-exposed group (Supplementary Fig. 5).

Altogether, the live animal imaging results indicate that the therapeutic window of engineered hMSCs in the wound beds of *db*/*db* mice is ~7–9 days, which coincides with the inflammatory and early tissue regeneration phases of wound healing^24^. We conclude that engineered hMSCs can survive and carry out transgene functions in the early phases of wound healing, despite the immune-competency of the animal model and the inflammatory environment. In addition, our observation that engineered Fluc MSCs cleared at different rates in wounds of pre-exposed *db*/*db* mice, in non-wounded *db*/*db* skin in the absence of wound-related inflammation, and in non-wounded immune-compromised NSG skin suggest that both the recipients’ immune system, including the adaptive immune system, and hMSC-intrinsic poor general survival in mouse skin affected such therapeutic window in diabetic mouse wounds. Having established the therapeutic window for engineered hMSCs in diabetic mouse wounds, we concluded that injected engineered hMSCs have potentials to carry out therapeutic functions during the inflammation and proliferation phases of wound healing without replacing the host cells long term in newly formed skin.

### hMSCs can be engineered to accelerate diabetic wound healing

**Fig. 4:**
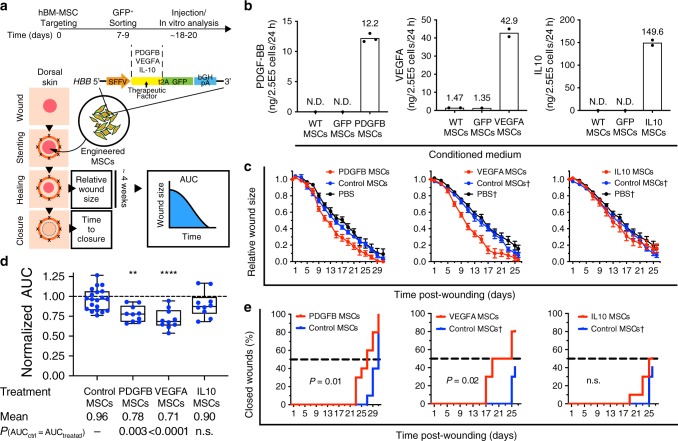
Engineered therapeutic factor-secreting hBM-MSCs improved kinetics of wound healing in acute diabetic wounds. **a** Excisional stented wounds created on the dorsal skin of hyperglycemic (>500 mg/dl blood glucose) *db*/*db* mice were treated with 2.5 × 10^5^ engineered therapeutic factor-secreting hBM-MSCs and imaged with digital photography every other day until complete closure. Relative wound size were determined by measuring the wound area relative to constant silicone ring area, and normalized to original wound size. Areas under the curve (AUC) were calculated for each individual wound. Time to complete closure was determined in 2-day intervals and unclosed wounds were excluded at the end of observation periods. **b** Bars represent average (*n* = 2 technical cell-plating replicates for all groups except PDGFB MSCs (*n* = 3 technical cell-plating replicates)) total level of therapeutic proteins secreted by 2.5 × 10^5^ engineered hMSCs, wild-type, and GFP-integrated controls over 24 h, as quantified by ELISA assay. **c** Dots and lines show changes in average relative wound size over time of engineered hBM-MSC treatments against PBS vehicle-treated and baseline MSC controls (biological replicate: *n* = 10 wounds per treatment group). Error bars represent standard error of mean. Daggers represent same control datasets. **d** Box and whiskers plots show AUC of individual wounds (*n* = 10 per treatment group), which were normalized against mean AUC of PBS controls (*n* = 10 PBS control wounds) within the same experimental runs. Minima and maxima are bounded by whiskers. Box lower bounds, centers, and upper bounds represent the 25th, 50th, and 75th percentiles, respectively. Mean AUCs of all treatment groups were different according to ordinary one-way ANOVA test (*p* < 0.0001). Mean AUCs of engineered hBM-MSCs treated wounds were compared to that of control hBM-MSCs-treated wounds using Dunnett’s test. Reported *p*-values were corrected for multiple comparison. Asterisks indicate level of significance (**p < 0.01; ****p < 0.0001). **e** Kaplan–Meier’s plots represent time to complete wound closure of engineered hBM-MSCs treated wounds, as compared to control hBM-MSC treatment. Chi-square *p*-values represent significant difference in time to closure between two groups according to Log-rank test. Daggers represent same control datasets. Source data are available in the Source data file.

To engineer hMSCs to treat diabetic wounds, we designed a modular safe harbor therapeutic protein overexpression cassette for integration at the *HBB* locus (Fig. 4a). Our design criteria geared toward elevated therapeutic protein production. We utilized growth factors and cytokines—PDGF-BB, VEGFA_165_, and IL-10—which are known to contribute to immune regulation and cellular migration and proliferation in wound healing^31–33^, and the bi-cistronic expression cassettes for *HBB* insertion containing reading frames of a synthetic growth factor cDNA and GFP cDNA (Fig. 4a, Supplementary Table 1). We successfully integrated the exogenous DNA sequence in the hBM-MSC genome with this vector design and purified targeted GFP^+^ cells by FACS (Supplementary Fig. 6). To confirm transgene function, we quantified levels of secreted growth factors in conditioned medium of therapeutic cassette-integrated hBM-MSCs (PDGFB, VEGFA, and IL-10 MSCs) as well as WT and GFP cassette-integrated hBM-MSCs (GFP MSCs). Although 2.5 × 10^5^ WT cells did not secrete detectable level of PDGF-BB over 24 hours, engineered PDGFB MSCs secreted 12 ng/day of PDGF-BB. The same number of VEGFA MSCs secreted 43 ng/day of VEGFA_165_—a 29-fold increase from WT. An equal number of IL-10 MSCs secreted 150 ng/day of IL-10 despite non-detectable WT baseline (Fig. 4b).

To assess therapeutic functions of engineered hMSCs, we utilized the excisional wound model in *db*/*db* mice to mimic human diabetic wound healing by re-epithelialization and granulation tissue formation with restrictive skin contraction^34^ (Fig. 4a). In this experiment, we created two 6-mm full-thickness punch biopsy wounds on the dorsum of 13–15-week-old mice and stented the wounds with silicone rings and sutures to prevent wound contraction (a closer mimic of human wound healing than the wound contraction mechanism that mice normally employ). We injected 2.5 × 10^5^ engineered hMSCs into each wound bed (*n* = 10 wounds per treatment group) shortly after wounding and imaged the wounds in 2-day intervals until closure. Wound sizes were calculated relative to the constant size of silicone rings and normalized to original wounds (day 0). At the selected cell dose, WT and GFP MSC controls yielded similar wound healing kinetics to vehicle (PBS) controls; however, we observed accelerated reduction of relative wound size over time in wounds treated with PDGFB MSCs and VEGFA MSCs, but not with IL-10 MSCs (Fig. 4c). The difference in wound healing rates can be observed starting at 7–9 days post wounding and treatment. At the end of the observation period, we graphically computed areas under the curve (AUCs) for each individual wound and normalized the values to the average AUC of PBS-treated wounds from the same experimental runs. We found that normalized areas AUCs for wounds treated with PDGFB MSCs (mean ± s.d. = 0.78 ± 0.10) and with VEGFA MSCs (0.71 ± 0.12), but not with IL-10 MSCs (0.90 ± 0.16), are significantly smaller than those of the control wounds (0.96 ± 0.14) that were treated with MSCs without growth factor hypersecretion (Fig. 4d). We also measured the time to complete closure of each wound in 2-day intervals. Similarly, the resulting time-to-closure functions for both the PDGFB and VEGFA MSC-treated wounds were significantly different from control groups (Fig. 4e). These results demonstrate that the integrated therapeutic gene cassettes enhanced efficacy of hBM-MSCs in diabetic wound healing by elevating secretion of growth factors in the wound bed. Given the therapeutic windows of 7–9 days as established in the previous section, we conclude that the presence of engineered MSCs that stably secreted PDGF-BB or VEGFA in the first week of healing was sufficient to accelerate healing of acute diabetic wounds over treatment with wild-type hBM-MSCs.

Having estimated a 9-day therapeutic window, we hypothesized that repeated treatment could extend therapeutic protein exposure and might further accelerate healing of the diabetic wounds. Upon observing the notable drop in luciferase activity around 7 days after Fluc MSC injection, we hypothesized that repeated injection of engineered hMSCs at 7 days post wounding may further accelerate healing. Following wound bed injection of 2.5 × 10^5^ VEGFA MSCs on the day of wounding, we repeated the injection at 7 days post wounding with either VEGFA MSCs or PDGFB MSCs. Nevertheless, the resulting healing kinetics appeared to be similar to those of single treatment with VEGFA at the day of wounding (Supplementary Fig. 7). We infer that the initial exposure to VEGFA MSCs sufficiently conditioned healing wounds toward complete closure, and because further treatment with VEGFA or PDGFB MSCs at 7 days post wounding had no additional benefit on healing kinetics in this animal model for 6-mm wounds, we speculate that early interventions with engineered hMSCs might be critical for accelerated healing.

### Engineered MSC-secreted GFs prompt wound cell proliferation

To observe local effects of secreted growth factors (GFs) on the wound tissue, we looked for evidence of PDGFB and VEGFA functions within histological sections from engineered MSC-treated skin. At the end of the healing kinetics observation period, we harvested punch biopsies of newly formed skin to observe its structure and composition.

**Fig. 5:**
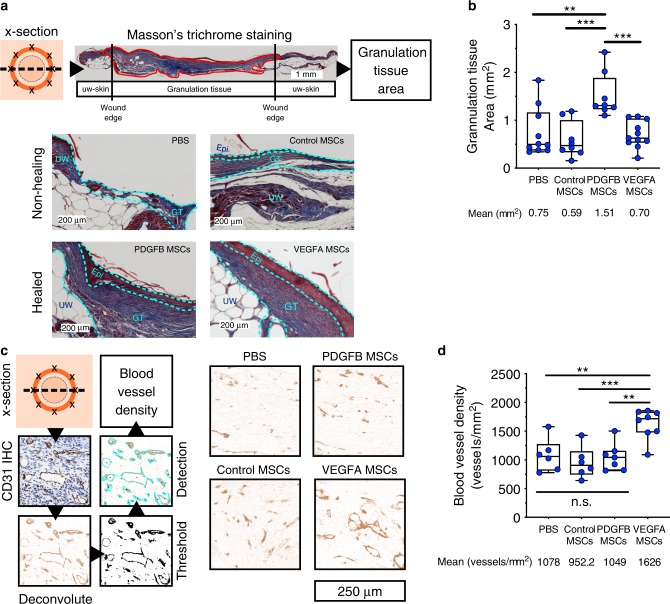
Growth factor activities of PDGFB and VEGFA MSCs influenced local changes in newly formed skin tissue. **a** Harvested tissues from 1-month-old wounds were cross-sectioned and stained with Masson’s trichrome dyes before light microscopy imaging. Amounts of granulation tissue were quantified as cross-section areas from high-resolution digital images. Representative images show impaired granulation tissue formation, which can be found in wounds from controls treatment groups, and mature granulation tissue in wounds from PDGFB MSC and VEGFA MSC treatment groups. Epi epithelium, GT granulation tissue, UW unwounded skin. **b** Box and whiskers plots show distributions of granulation tissue areas measured from cross-sections of individual wounds from PBS and hMSC controls, PDGFB MSC, and VEGFA MSC treatment groups (*n* = 10, 8, 8, and 10 wounds, respectively). Minima and maxima are bounded by whiskers, lower bounds, centers, and upper bound of boxes represent the 25th, 50th, and 75th percentiles, respectively. Differences of means were tested by ordinary one-way ANOVA and *p*-values were computed using Tukey’s multiple comparisons test. Asterisks indicate *p*-values (*p < 0.05; **p < 0.01, n.s.: *p* > 0.05). **c** (Left panel) Harvested tissues from one-month-old wounds were cross-sectioned (x-section) and stained with anti-mouse CD31 antibody. Stained blood vessels were detected from digital staining images using ImageJ software. Blood vessel density was calculated from whole granulation tissues. (Right panel) Representative blood vessel images from controls, PDGFB MSC, and VEGFA MSC treatment groups are shown. **d** Box and whiskers plots show distributions of blood vessel densities measured from cross-sections of individual wounds from PBS and hMSC controls, PDGFB MSC, and VEGFA MSC treatment groups (*n* = 6, 6, 7, and 8 wounds, respectively). Minima and maxima are bounded by whiskers, lower bounds, centers, and upper bound of boxes represent the 25th, 50th, and 75th percentiles, respectively. Differences of means were indicated by ordinary one-way ANOVA and *p*-values were computed using Tukey’s multiple comparisons test. Asterisks indicate *p*-values (**p < 0.01; ***p < 0.001, n.s.: *p* > 0.05). Source data are available in the Source data file.

PDGF-BB is known to have roles in recruitment and proliferation of fibroblasts in the granulation tissue, which leads to collagen deposition to increase structural integrity of the wounds. Therefore, to observe effects of PDGF-BB MSCs, we quantified the amount of granulation tissue in Masson’s trichrome stained sections of wounds from control and effective treatment groups. We observed collagen deposition in the newly formed granulation tissue and measured total cross-section area of collagenous granulation tissue under the epidermis and between two wound edges (Schematics shown in Fig. 5a). We observed thickening of the collagenous granulation tissue in PDGFB MSC-treated wounds, whereas incomplete granulation tissue formation and epithelialization were observed in control treatment groups (Fig. 5a). We quantified cross-sectional granulation tissue area in digital images of healed wound sections and found that PDGFB MSC-treated wounds, have significantly increased cross-sectional area of total granulation tissue over vehicle control (*p*-value < 0.01, ordinary one-way ANOVA with Tukey’s multiple comparison test), control MSC-treated wounds (*p* < 0.001 ordinary one-way ANOVA with Tukey’s multiple comparison test), and VEGFA MSC-treated wounds (*p* < 0.001 ordinary one-way ANOVA with Tukey’s multiple comparison test) (Fig. 5b). These results support the notion that accelerated closure of PDGFB MSC-treated wounds are due to the accelerated granulation formation specifically via PDGFB signaling that has a known role in recruiting and promoting proliferation and collagen production of nearby fibroblasts.

VEGFA is known for its roles in directing endothelial cell migration and vascularization during wound healing. To observe wound bed vascularization, we detected blood vessels in wound sections with marker CD31 immunohistochemistry and quantified blood vessel density using built-in digital image analysis tools on the ImageJ software. We performed color deconvolution to extract the 3,3′-diaminobenzidine (DAB) staining of mouse CD31 and used a particle analysis algorithm to detect blood vessels on thresholded DAB images of granulation tissues (Fig. 5c). The resulting particle counts normalized over granulation tissue area revealed that VEGFA MSCs treated wounds became significantly more vascularized, with a 1.5-1.7 fold increase in overall blood vessel density over PBS- (*p*-value < 0.01, ordinary one-way ANOVA with Tukey’s multiple comparison test), control MSC- (*p*-value < 0.001 ordinary one-way ANOVA with Tukey’s multiple comparison test), and PDGFB MSC-treated wounds (*p*-value < 0.01 ordinary one-way ANOVA with Tukey’s multiple comparison test) (Fig. 5d). The higher degree of neovascularization in the wound bed of VEGFA-treated wounds suggest specific VEGFA signaling in the local tissue.

In sum, improvements observed in granulation tissue formation and blood vessel density for treated wounds are consistent with in vivo imaging results that showed engineered hMSCs expressing transgene functions locally at the injection site. We conclude that PDGFB MSCs and VEGFA MSCs exerted their therapeutic effects locally at the injection site. Further, the difference in effects of secreted growth factors in the wound bed of PDGFB and VEGFA MSC-treated wounds suggest that the two therapeutic MSC lines addressed different aspects of wound healing and may be combined to achieve synergistic effects. However, in wounds treated with 2.5 × 10^5^ engineered hMSCs consisted of a 1:1 ratio of PDGFB MSCs and VEGFA MSCs, we did not observe further acceleration of healing kinetics beyond VEGFA MSC treatment alone, or further increases in granulation tissue formation and blood vessel density (Supplementary Fig. 8). We note that the cell combination still significantly accelerated healing when compared to vehicle control. The results suggest that different therapeutic factors may affect the healing kinetics of wound healing at varying degrees.

### VEGFA MSCs function in wound-in situ crosslinked hydrogel

**Fig. 6:**
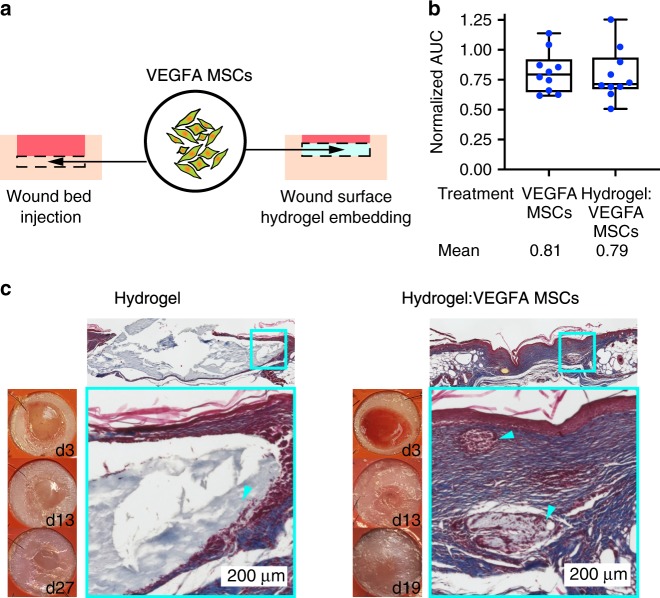
Engineered hMSCs are compatible with hydrogel scaffold treatment for wound healing. **a** VEGFA MSCs were delivered by injection in the wound bed or by HyStem®-HP hydrogel embedding on top of the wound bed on the day of wounding. **b** Box and whiskers plot shows AUC of individual wounds, which were normalized against mean AUCs of PBS controls (biological replicates: *n* = 10 per treatment group). Minima and maxima are bounded by whiskers, lower bounds, centers, and upper bound of boxes represent the 25th, 50th, and 75th percentiles, respectively. Mean AUCs of all treatment and control groups were different according to ordinary one-way ANOVA test (*p* < 0.0001). Plotted treatment groups have no significant differences in AUCs. **c** Wound images show appearances of wounds treated with HyStem®-HP hydrogel alone (label: “Hydrogel”) and embedded VEGFA MSCs (label: “Hydrogel:VEGFA MSCs”) over the course of observation period. Masson’s trichrome stained cross-section images show structure of granulation tissues at the end of observation period. Cyan boxes indicate location of high-resolution images. Cyan arrowheads indicate residual Hystem®-HP hydrogel. Source data are available in the Source data file.

Extracellular matrix-like hydrogels can be used for scaffolding and growth factor encapsulation to promote wound healing^35^. Therefore, we reasoned that engineered hMSCs can serve as the source of growth factor from within the scaffold. To demonstrate compatibility of engineered hMSCs with tissue engineering approaches for wound healing, we embedded VEGFA MSCs in in situ crosslinked HyStem®-HP hydrogel comprising of crosslinked hyaluronan, heparin, and denatured collagen above the wound bed (Fig. 6a). Although the hydrogel alone did not improve healing kinetics, we found that embedded VEGFA MSCs above the wound bed yielded similar healing kinetics to injected VEGFA MSCs in the wound bed (Fig. 6b, Supplementary Fig. 9). We conclude that the secreted VEGFA in the scaffold can affect the surrounding wound environment to accelerate healing. Interestingly, from harvested wound sections, we observed slow degradation of HyStem®-HP hydrogel when applied alone despite some degrees of surface granulation tissue formation and epithelialization, but we saw consistent cellular infiltration and degradation of the hydrogel in the presence of VEGFA MSCs (Fig. 6c). This difference in degradation rate and cellular infiltration suggests that hMSCs helped promote rapid degradation and replacement of the scaffold with granulation tissue, possibly through migration of endothelial cells into the scaffold. These results in this section support the notion that engineered hMSCs can be incorporated with existing tissue engineering approaches in wound therapy.

## Discussion

Despite a limited number of advanced clinical trials and approved hMSC products, significant progresses have been made by the scientific community to establish safety profiles and pipelines for clinical-grade manufacturing^4,10,36^. Such achievements for MSC-based therapy can advance further with a platform to precisely genetically engineer more therapeutically potent hMSCs or to study their mechanisms of action. In this study, we established conditions that maximize targeted integration in ex vivo hMSCs using MS-modified sgRNA, Cas9 nuclease, and AAV6-based homologous recombination templates. Our genome-editing tools led to gene disruption and targeted integration of up to 3.2 kb of DNA with stable transgene expression in hMSCs from multiple tissue sources without disrupting their in vitro characteristics. In the murine *db*/*db* diabetic wound healing model, we demonstrated transient survival of engineered hMSCs and a window of integrated transgene function in the wound tissue and showed enhanced therapeutic functions of engineered hMSCs, which can exert therapeutic effects locally through known functions of integrated therapeutic genes, and can be integrated with tissue engineering-based treatment. The most distinct advantages of our Cas9-AAV6 platform lie in its compatibility with hMSC manufacturing, its versatility, and its potentials for therapeutic applications.

Our delivery methods and targeting procedures can be integrated seamlessly into established hMSC manufacturing protocols. The genome editing itself can be done in a short, two-step process. Our optimized, non-toxic electroporation condition can improve yields of engineered hMSCs by maximizing delivery of nucleic acids, while eliminating excessive toxicity seen in previous reports^37,38^. Of note, additional toxicity due to a double-stranded break response can be minimized when using RNP nuclease^29^. Similar to our previous findings, electroporation has synergistic effects on hMSC transduction with AAV6^39^. Interestingly, this phenomenon eliminates the need for high-density hMSC culture for AAV transduction^15^. Such rapidly initiated AAV6 contact favors low-density monolayer expansion, which is optimal for hMSC doubling and preservation of their potentials^40^. This hit-and-run DSB induction and HR-based gene integration can produce gene knockout lines within 4 days and gene-integrated products in 4–7 days, with the option of enrichments with selectable markers. The resulting engineered products can also be further expanded for several passages to maximize yield.

Our genome-editing platform is also versatile in its compatibility with different hMSC types, genome coverage, magnitude of manipulations, and repertoire of applications. While originally described as colony-forming stromal cells of the bone marrow^41^, in their most permissive definition, MSCs are fibroblast-like plastic-adherent stromal cells that can be isolated from multiple tissues and can have different properties^28,42^. Our ability to manipulate MSCs from different sources can be useful for a large number of clinical indications and valuable for many investigators in the extensive MSC research community. The use of guide RNAs and Cas9 simplifies nuclease designs for sequence-specific DNA recognition over engineered ZFNs or TALENs. In addition, the highly frequent occurrence of the NGG motif for Cas9 recognition is responsible for its wide target coverage^43^. By using different sets of sgRNA and homology arms in AAV6 donors, we could generate indels and integrate transgenes, potentially at all Cas9-targetable sites. In addition to our Cas9/AAV system, previous studies by Xu et al.^44^ showed moderate indel-generating DBS activity of Cas9 RNP in human MSCs and possible HR-based single-nucleotide deletion with Cas9 RNP and single-stranded oligodeoxynucleotide (ssODN) template using tube electroporation. Similar non-viral approach was shown by Roth et al.^45^ in primary T cells in which targeting with Cas9 RNP and ssODN or double-stranded DNA (dsDNA) can lead to efficient integration of up to 1.5 kb into the genome. The non-viral methods described by Xu and Roth are currently limited by insertion size. Insert size of ~1.5 kb (not including ~600 kb of homology arms) appear to be an upper limit of the system due to synthesis technique of ssODN and dsDNA, where commercial source only offer up to 2 kb synthesis in small amounts. In contrast, AAV allows ~3.5 kb of insert with single donor targeting, and can be obtained commercially through plasmid cloning and AAV production services. In addition, our method has been used in other primary stem and progenitor cells to modify the genome at the single-nucleotide resolution^23^, in multiplexed, multiallelic and multigenic integrations^46^, and in serial integration of large inserts^47^. By establishing such a platform for hMSCs, we offer a tool that can alter genomes ranging from single base substitution up to 6.5 kb insertion and is amenable to diverse customizations. These entail the capability to create or rescue mutations, manipulating endogenously expressed genes and their expressions, and conferring transgene expression from safe harbors such as *HBB* with rooms for additional regulator elements or safety switches. For ease of application and production, bioinformatic and commercial services are available for guide RNA and repair template design, and both laboratory and clinical-grade reagent production. We note that the specificity profile for the gRNA targeting *HBB* has been previously described, and that its potential off-targets have no roles in hMSC functions^23,30,48,49^. The genome-editing technique is useful not only for cell therapies, but also for genetic studies, disease modeling and correction, drug screening, and top-down synthetic biology to retrofit novel MSC functions^50^. Furthermore, as transgene expression can be carried from engineered MSCs into their differentiated descendants, our platform can also be used in MSC-based tissue engineering. For therapeutic purposes, our modular cassette designs accommodate changes of cassette elements, as shown by using different therapeutic protein coding sequence in this study. Clinically compatible elements such as promoters and selection markers can be customized based on specific needs of production or mode of therapy. We note that customizations include choice of integration sites that can be controlled for precise level of gene expression and interactions with other genomic loci.

Engineered therapeutic protein-expressing MSCs will be generally valuable for short-term treatments of injury without replacing the recipient tissue. Although we previously showed that engineered mouse fibroblasts secreting PDGF-BB can improve wound healing^51^, the retention of the engineered fibroblasts and their PDGF-BB production long term may have further oncogenic effects. The FDA temporarily issued warning on long term use of Regranex® as it has potentials to cause mortality secondary to malignancy^52^. Comparing to recommended dosage of Regranex® in human per cm^2^ of wound area, we achieved healing in mice with an estimated PDGF-BB secretion (in vitro) of ~12 ng/day by 2.5 × 10^5^ PDGFB MSCs, which is drastically lower than 62.5 mg/day used with the topical gel for a comparable wound size (according to FDA label for Regranex®). In addition, engineered hMSCs will be suitable for treatment of tissues where resident cells cannot be isolated or manipulated in culture, and where short-term interventions are needed. For clinical applications, while random integration by retroviral/lentiviral vectors seem more accessible for clinicians, random transgene-integrated cells will require extensive tumor-toxicity study for each clinical application and production round due potentials for oncogenic activation and varying level of transgene expression which depends on integration sites. Further, the Cas9-AAV platform has unique potentials for advanced cell-based therapy, where immune compatibility genes such as *B2M* and *HLA* genes, instead of MSC-specific safe harbor such as *HBB*, could be disrupted by transgene integration to reduce immune clearance of hMSCs in therapy and enable utilization of off-the-shelf allogeneic MSC products.

Lastly, it is important to note that our understanding of native clinical properties of hMSCs and insights into specific therapeutic needs of each disease will help determine effective engineering strategies for most efficacious MSC-based treatments using our platform. Although infused MSCs are not known to elicit severe immune responses in patients, there are no conclusive evidence on effectiveness and differential survival of allogeneic or autologous MSCs^2,53^. Repeatable MSC-based treatment for recurring issues like chronic wounds will rely on immune compatibility of autologous cells or immune escape functions of allogeneic cells, which can be native or engineered by knocking out immune histocompatibility genes such as *B2M* and *HLA* genes^54^. Regardless, both autologous and allogeneic MSC therapies can utilize the engineering platform to enhance therapeutic functions. The short ex vivo exposure to Cas9 and AAV6 eliminate additional potential cytotoxic immune responses to genome-editing components in the engineered cells. Moreover, understanding of biodistribution, kinetics, and mechanism of action of engineered MSCs in human following local and systemic delivery in different recipient tissues will dictate potential applications and engineering strategies for survival and temporal controls. For example, improvements in wound healing by both PDGFB- and VEGFA-hypersecreting hMSCs highlight possibilities for combinatorial therapy. The modularity of our genetic engineering platform allows for complex treatments using mixtures of engineered MSCs or engineered multi-functional MSCs with different therapeutic molecules to address multiple mechanistic and temporal aspects of diseases or injuries. Our results suggest that the understanding of the complex interplay between growth factors at different stages of wound healing will dictate suitable dose, combination, and timing of engineered MSC treatments. Given the complexity of the wound healing process and other tissue injuries, our results further highlight that it is necessary to rationally and experimentally determine the suitable total dosage and composition of the multiple therapeutic proteins in order to take full advantage of the platform and further accelerate healing kinetics.

In conclusion, we envision that this genetic engineering platform will be of value to the broad MSC research community, to help advance our basic understanding of MSC biology and to extend the scope and impact of MSC-based therapeutics.

## Methods

### Mesenchymal stromal cells

Frozen hBM-MSCs from healthy donors were obtained through the Institute of Regenerative Medicine—University of Texas A&M, the laboratory of Dr. Ravindra Majeti (Stanford University), and All Cells Inc. Frozen hAD-MSCs were purchased from Life Technologies (Carlsbad, CA), Millipore Sigma (Burlington, MA), and iXCells Biotechnologies (San Diego, CA). Fresh CD34^−^ cord blood mononuclear cells (MNCs) were acquired through the Binns Program for Cord Blood Research at Stanford University approved by the Stanford Institutional Review Board. Frozen samples were thawed according to standard protocols. For hUCB-MSCs, fresh CD34^−^ MNCs from cord blood were seeded at 1.0 × 10^6^ cells/cm^2^ density and cultured for 21–30 days until colonies formed. All hMSCs were expanded in culture by seeding at 60 cells/cm^2^ density in complete culture medium consisted of α-modified minimum essential medium (α-MEM, Gibco, Invitrogen, Carlsbad, CA), supplemented with 16.7% (v/v) fetal bovine serum (Gibco), 2mM l-glutamine, and 1% penicillin/streptomycin cocktail. Cells are subcultured when confluency reached 80–90%.

### AAV6 vectors

All AAV6 vectors were prepared from co-transfection of vector plasmids and pDGM6 helper plasmids were packaged in PEI into 293T cells. 293T cells were cultured in monolayers in DMEM media (Corning, Corning, NY) supplemented with 10% fetal bovine serum and 1% penicillin/streptomycin cocktail to reach 90% confluency. Then, the transfection mixture—which contains a 6:22 vector-to-helper plasmid ratio (w/w) and a 4:1 PEI-to-DNA ratio (w/w) in Opti-MEM transfection medium—were added to the 293T culture so that an equivalent of 6 μg of vector DNA is added to one 15-cm dish. At 48–72 h later, cell pellets were collected by scraping and centrifugation. Cell lysates were obtained after three freeze–thaw–trituration cycles and cellular debris were discarded. Lysates were treated with TurboNuclease (ThermoFisher Scientific, Waltham, MA) for 45 min and fractionated on iodixanol density gradient containing 15, 25, 40, and 58% iodixanol layers by ultracentrifugation at 237,000×*g* for 2 h at 18 °C. Packaged capsids were extracted from the 40–58% iodixanol interface and dialyzed twice in PBS (Corning) and once in 5% d-sorbitol (Sigma-Aldrich, St. Louis, MO) in PBS. Pluronic acid (ThermoFisher Scientific) was added to the dialyzed product to a 0.001% final concentration. AAV6 was stored at −80 °C. AAV concentration was determined by a qPCR assay using inverted terminal repeat (ITR)-binding probe (forward primer: 5′-GGAACCCCTAGTGATGGAGTT-3′; reverse primer: 5′ -CGGCCTCAGTGAGCGA-3′, and ITR probe: 5′ -CACTCCCTCTCTGCGCGCTCG-3′ (6-FAM/ZEN/IBFQ)).

### Ex vivo genome editing of hMSCs

Human MSCs were cultured to 80–90% confluency before being trypsinized (TrypLE Express) and resuspended to 5000 cells/µl in Opti-MEM® I (Invitrogen). Nucleases and sgRNA (Synthego, Menlo Park, CA; TriLink Biotechnologies, San Diego, CA) were mixed in as All-RNA cocktail (final concentration: 0.1 µg/µl MS-modified sgRNA with 0.15 µg/µl Cas9 mRNA (5′ MeC, ψ, TriLink Biotechnologies)) or precomplexed RNP (10 min at room temperature, 1:2.5 molar sgRNA-to-Cas9 ratio, final concentration: 0.3 µg/µl Cas9 protein (Integrated DNA Technologies, Coralville, IA)). Then, hMSC-Nuclease mixtures were electroporated with pulse code CM-119 on the Lonza 4D Nucleofector (Lonza Group AG, Bazel, Switzerland) and immediately diluted with 2× volume Opti-MEM® I. If repair template is required, 1.0 × 10^5^ vector genomes/cell of AAV6 (or as otherwise indicated) were added to the mixture after dilution step. The electroporated hMSC-AAV6 mixtures were then incubated at 37 °C for 15 min before seeding at ~220 cells/cm^2^ in complete culture medium. Medium was replaced 48 h after targeting.

### Flow cytometry and FACS

Efficiency of fluorescence gene integration was measured by flow cytometry at different timepoints after targeting. As shown in Supplementary Fig. 3, Live MSC population (7-AAD^−^ or PI^−^) of single hMSCs were analyzed for composition of GFP^+^ fraction. For purification, GFP^high^ or GFP^+^ populations obtained at one passage post targeting were sorted on a BD FACS II Aria SORP (BD Biosciences, San Jose, CA) and expanded for further analyses and uses in animal models.

### Molecular quantitation of indel and gene integration

Frequencies of indel mutations were measured by sequence trace analysis (TIDE or ICE analysis^55,56^). Briefly, genomic DNA was isolated from populations of hMSCs electroporated with nucleases after 4 days in culture. The region surrounding DSB site was amplified by PCR assays and submitted for Sanger sequencing. Compositions of −10 nt deletion to +10 nt insertion (symbols: (−i) deletion of i nt around cut site, (+i) insertion of i nt around cut site) were computationally derived using the TIDE software (https://tide.nki.nl/) or ICE software (https://ice.synthego.com). Total integration frequencies were quantified by a digital droplet PCR assay, where absolute quantity of amplified and detected integration junction was normalized to numbers of amplified and detected endogenous *CCRL2* alleles^39^. Frequency of mono-allelic and bi-allelic integration were determined by clonal analysis where limiting dilution of sorted GFP^+^ hMSCs were plated at densities between 10 to 30 cells/cm^2^ culture surface, allowed to form colonies, which were then harvested with cell scrapers. Genomic DNA of single hMSC colonies were isolated and subjected to PCR amplification (forward primer: 5′-TAGATGTCCCCAGTTAACCTCCTAT-3′; reverse primer: 5′-TTATTAGGCAGAATCCAGATGCTCA-3′) and genotypes were determined by gel-electroporesis separation of integrated and non-integrated amplicons by size.

### Immunophenotyping

Immunophenotyping was performed to determine cell surface marker profiles of hMSCs before and after gene targeting according to ISCT consensus^28^. Cells were trypsinized and stained to analyze with flow cytometry, with the following monoclonal antibodies (and clone IDs) from BioLegend Inc. (San Diego, CA): CD105-PE/Cy7 (43A3), CD73-APC (AD2), CD90-PerCP/Cy5.5 (5E10), CD14-Pacific Blue (M5E2), CD19-Pacific Blue (SJ25C1), CD34-Pacific Blue (581), CD45-Pacific Blue (2D1), and HLA-DR-Pacific Blue (L243). All antibodies were used at 1:100 dilutions for staining.

### Tri-lineage differentiation

Wild-type and gene-integrated hMSCs were cultured in StemPro adipogenesis, osteogenesis, or chondrogenesis induction media according to manufacturer protocols (ThermoFisher Scientific). Adipogenic differentiation cultures were stained for lipid droplets with BODIPY558/568-C12 or LipidTOX-Red dye (ThermoFisher Scientific) at 7–14 days of culture. Osteogenic differentiation cultures were stained with Alizarin Red (Millipore Sigma, Burlington, MA) solution for deposited calcium after 21 days of culture. Chondrocyte pellets were stained with Alcian Blue (Sigma-Aldrich) for proteoglycans and embedded in optimal temperature cutting compound (Fisher Scientific) and frozen sections were obtained for microscopy. Fluorescence and brightfield images of MSCs in culture vessels were obtained with ThermoFisher Scientific EVOS FL imaging system for GFP and fluorescent dye visualization. Light microscopy for colored dye stained osteogenesis cultures and frozen chondrocyte sections were obtained with Keyence BZ-x800 microscope system (Osaka, Japan).

### Animals

All *db*/*db* mice were purchased from the Jackson Laboratory (Bar Harbor, ME) and maintained in Stanford University’s Comparative Medicine Pavillion and all NSG mice were maintained in colonies under the care of the university’s Veterinary Service Center staff in the SIM-1 barrier facility. All mice were experimented under protocols approved by Stanford University’s Administrative Panel on Laboratory Animal Care.

### In vivo imaging

FACS sorted and expanded Luciferase^+^GFP^+^ hBM-MSCs were injected into the wound beds or non-wounded skin of anesthetized *db*/*db* mice or NSG mice (2.5 × 10^5^ cells/mouse) on the day of wounding unless otherwise indicated. At different timepoints post hMSC injection, luciferase activities were detected in vivo. Mice were injected intraperitoneally with d-Luciferin (0.15 g luciferin per 1 g bodyweight) and imaged 10 min after with IVIS-100 or IVIS Spectrum imaging system (Perkin Elmer, Waltham, MA), or with Ami Imager (Spectral Instruments Imaging, Tucson, AZ, USA) based on instrument availability in different animal housing facilities. Images were obtained and analyzed using manufacturers’ software (Perkin Elmer: LivingImage; Spectral Instruments Imaging: Aura). Bioluminescence was quantified using the LivingImage software (Perkin Elmer).

### ELISA

In 12-well plates, 1.0 × 10^5^ hMSCs per well were seeded in complete culture medium overnight. Overnight medium was replaced with 1 mm of fresh medium and cells were further incubated for 24 h before conditioned medium was obtained. As needed, conditioned medium was stored at −80 °C before use. Growth factor (PDGF-BB, VEGFA, or IL-10) concentration of was determined using the RayBiotech (Peachtrees Corners, GA) ELISA kits per manufacturer protocols. Complete medium, and conditioned medium from WT and GFP MSCs were assayed without dilution. Conditioned medium from PDGFB MSCs, VEGFA MSCs, and IL-10 MSCs were diluted in assay buffer at 1:10, 1:20, and 1:1000 ratio, respectively. Spectral profile of colorimetric solutions were obtained and quantified using the BioTek Synergy H1 microplate reader and BioTek Gen5 software (Winooski, VA). Amount of growth factors/cytokines secreted by 2.5 × 10^5^ hMSCs were linearly scaled from amounts measured from 1.0 × 10^5^ cells.

### Diabetic wound assay

Thirteen to fourteen-week-old *db*/*db* mice with blood glucose levels greater than 500 mg/dl were depilated and two 6-mm full-thickness wounds extending through the panniculus carnosus were made at the same level on the dorsum of the mice on either side of the midline according to an established model^57^. A donut-shaped silicone splint with a 10-mm diameter was centered around the wound and affixed to the skin using adhesive (Krazy Glue) and interrupted 6-0 nylon sutures (Ethicon, Somerville, NJ). For treatment, mice were randomized to different treatment groups. Unless otherwise stated, 2.5 × 10^5^ engineered hMSCs suspended in PBS were directly injected into the subcutaneous layer of the wound bed. Wounds were covered with Tegaderm (3M, Maplewood, MN), which was replaced every other day, at which times digital photographs of the wounds were taken until wounds are fully re-epithelialized. Wound areas were digitally measured relative to inner area of silicone rings, using Adobe Photoshop CS6 (San Jose, CA), and normalized to original wound areas.

### Histology

At the end of diabetic wound assay, wound tissues were harvested with 8-mm punch biopsy tool and half-sections were embedded in FFPE. Masson’s trichrome staining and immunohistochemistry (IHC) was performed on 8-mm sections. Digital images of Masson’s trichrome stained slides were obtained using Keyence BZ-x800 microscope system and quantified using Adobe Photoshop software. Mouse CD31 IHC and whole slide digital scanning were performed by Histowiz Inc (Brooklyn, NY). The resulting H-DAB staining images were analyzed using ImageJ software functions. Eight-bit DAB staining areas were obtained using Color Deconvolution function, then five-level thresholded images were subjected to Analyze Particle function to obtained average total blood vessel count. Blood vessel densities were calculated against total areas of granulation tissue.

### In situ hydrogel embedding of hMSCs

HyStem®-HP hydrogels (ESI BIO, Alameda, CA) were prepared according to manufacturer protocol. Briefly, degassed water-dissolved Heprasil®, Gelin-S®, and Extralink® were mixed at a 2:2:1 ratio and used to resuspend a pellet of hMSCs to a concentration of 2.5 × 10^4^ cells/µl within 10 min. The liquid mixture was applied on top of wound beds underneath Tegaderm dressing and allowed to solidify in place. Dressings were changed again at 3 days post wounding.

### Reporting summary

Further information on research design is available in the Nature Research Reporting Summary linked to this article.

## Supplementary information

Supplementary Information

Reporting Summary

## Data Availability

All data that support conclusions stated are included in the manuscript. The source data underlying Figs. 1c, d, 2b, c, 3c, d, 4b–e, 5b, d, and 6b and Supplementary Figs. 1a–d, 2b–f, 3b, d, g, 5b, c, 7a–e, 8b–d, and 9a, b are provided as a Source Data file. Additional relevant data can be obtained from the authors upon reasonable request.

## Source data

Source Data
