# Supplementary material for: Cas9-AAV6-engineered human mesenchymal stromal cells improved cutaneous wound healing in diabetic mice
Source: Nat Commun. 2020 May 18;11:2470. doi: 10.1038/s41467-020-16065-3 (PMC7235221; doi:10.1038/s41467-020-16065-3)
Supplement: Supplementary file 1 — Supplementary Information [file 41467_2020_16065_MOESM1_ESM.pdf]

Cas9/AAV6-Engineered Human Mesenchymal Stromal Cells Improved Cutaneous Wound Healing in Diabetic Mice

Srifa et al.

Correspondence should be addressed to Matthew Porteus (mporteus@stanford.edu)

Lorry I. Lokey Stem Cell Building (SIM1)

265 Campus Drive, Room G3045,

Stanford, CA 94305, USA

Supplementary Table 1 | Information on sgRNAs and AAV6 donor repair templates

Target	Guide Sequence (PAM)	LHA (bp)	Insert (bp)	RHA (bp)
HBB	CTTGCCCCACAGGGCAGTAA(CGG)	538	SFFV-TurboGFP-bGH pA (1511)	420
			SFFV-PDGFB-T2A-TurboGFP-bGH pA (2297)	
			SFFV-VEGFA$_{165}$-T2A-TurboGFP-bGH pA (2147)	
			SFFV-IL10-T2A-TurboGFP-bGH pA (2108)	
			SFFV-Fluc-T2A-TurboGFP-bGH pA (3221)	
CCR5	GGCAGCATAGTGAGCCCAGA(AGG)	797	SFFV-Citrine-WPRE-bGH pA (2095)	800
RANKL	ATTCTATTAGGATCCATCTG(AGG)	487	SFFV-TurboGFP-bGH pA (1511)	556

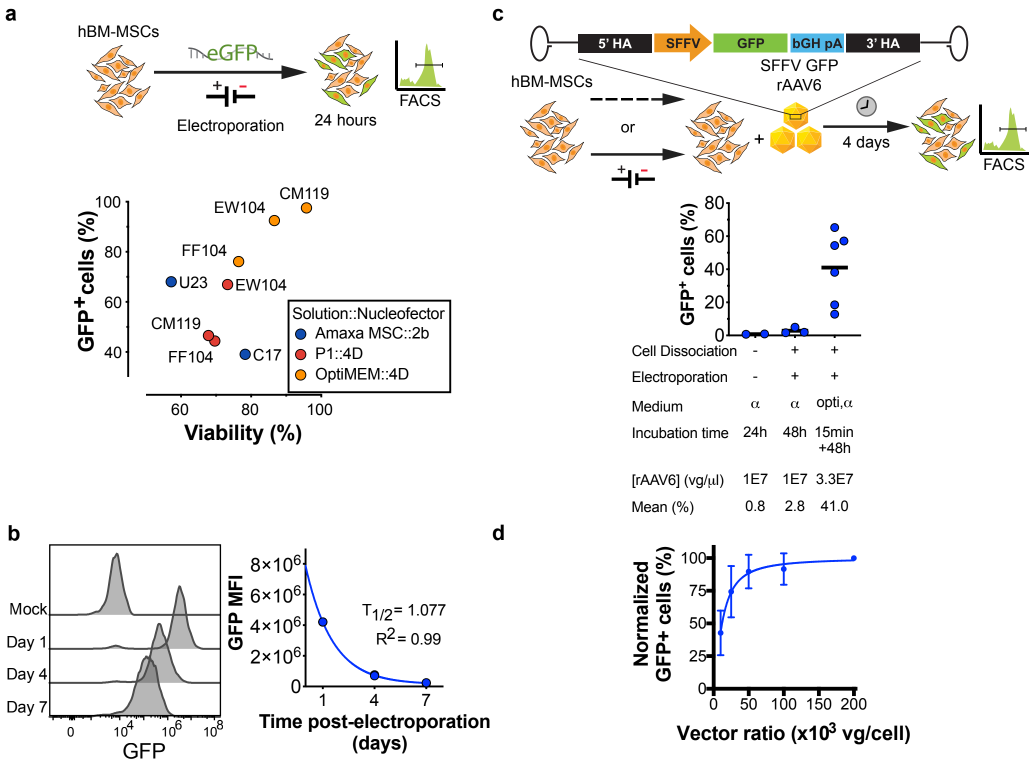

Supplementary Figure 1 | Electroporation and electroporation-aided transduction (EAT) of AAV6 vectors efficiently deliver nucleic acids into hBM-MSCs

a Synthetic eGFP mRNAs were electroporated into hBM-MSCs using different combinations of pulsing programs and suspension buffers on the Lonza 2b or 4D Nucleofector system. Plot shows distribution of cell recovery and GFP$^+$ cell frequencies at 24 hours post-electroporation for each program-buffer-nucleofector combination (Technical replicate: n = 2). **b** (left) Staggered FACS histograms show a representative distribution of GFP intensity in electroporated hMSC population (program: CM-119; solution: Opti-MEM®; Nucleofector: 4D) at indicated timepoint post-electroporation. (right) Plot shows curve fit of mean fluorescent intensities of GFP$^+$ cell population following eGFP mRNA electroporation (Technical replicate: n = 2). **c** hBM-MSCs were transduced using various protocols with AAV6 vectors harboring a GFP overexpression cassettes (HA: homology arm of HBB locus). Plot shows frequencies of GFP$^+$ cells for each biological replicate at four days after initial AAV6 exposure. α: alpha-MEM-based complete culture medium; Opti: Opti-MEM® (used during 15-minute incubation immediately after electroporation) (From left column to right) Biological replicates, n = 3, 3, and 6, respectively. **d** Three biological replicates of hBM-MSCs were transduced by the AAV6 vector by EAT at different vector-to-cell ratio. Plot shows curve fit of the resulting frequencies of transduced GFP$^+$ cells normalized to maximal transduction frequency for each donor. (R^2 = 0.73) Dots and error bars represent average normalized cell frequencies and standard deviation.

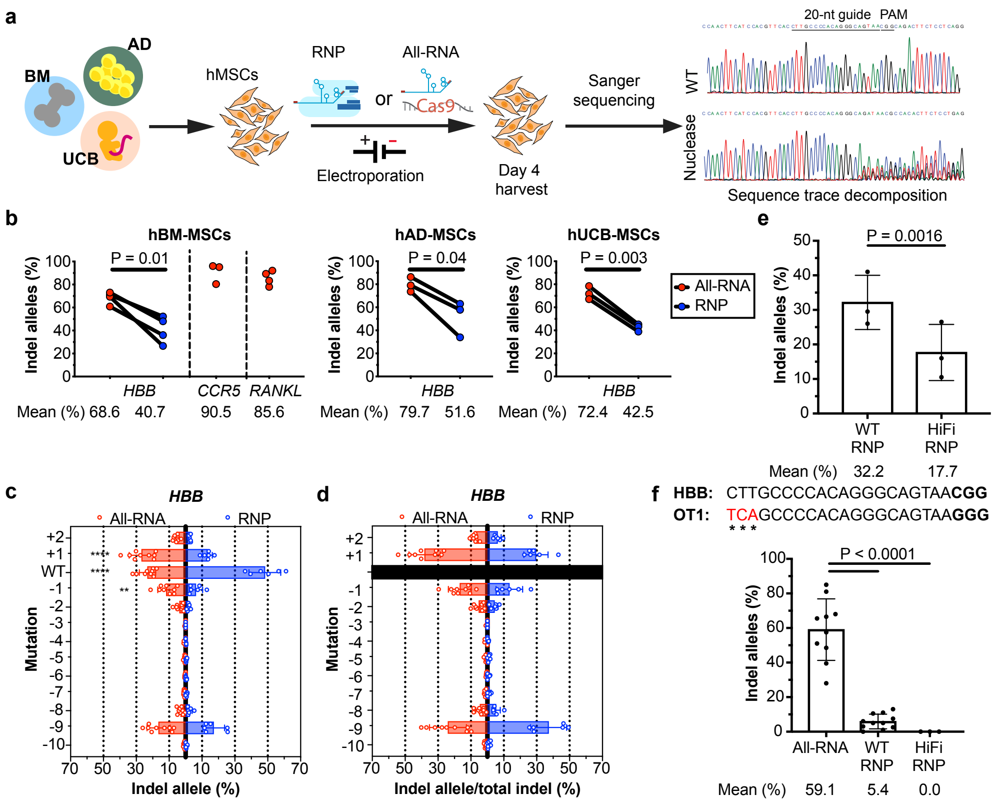

Supplementary Figure 2 | Targeting of the HBB locus with Cas9 nuclease yielded semi- predictable distribution of indel alleles

a Four days after electroporation with All-RNA nuclease cocktails or RNP complexes, bulk genomic DNA of hMSCs were subjected to sequence trace analysis for indel quantification. Representative images show Sanger sequencing chromatograms generated by PCR amplification around the *HBB* targeting site from genomic DNA of wildtype cultured hMSCs and nuclease electroporated hMSCs. Heterogeneity of sequence can be observed around the cut site in nuclease-treated genomic DNA. b Total indel frequencies generated in hBM-MSCs (biological replicates: n = 4), hAD-MSCs (n = 3), and hUCB-MSCs (n = 3) with All-RNA cocktails and RNP complexes are represented in dot plots for indicated gene loci (*HBB*, *CCR5*, and *RANKL*). Connected dots represent hMSCs from the same human donors. Mean indel frequencies were compared between two modes of nuclease delivery and two-tailed p-values from paired t-test are shown. **c** Dots and bars show percentage of indel sizes generated by Cas9-mediated DSB around the HBB break site for each biological replicate of hBM-MSCs and average values, respectively. Minus and plus signs represent deletion and insertion mutations, respectively. (All-RNA) n = 9; (RNP) n = 6. Error bars represent standard deviation. **d** Signature of indel frequencies normalized to total indel frequencies is shown. (All-RNA) n = 9; (RNP) n = 6. Error bars represent standard deviation. **e** Dots and bars represent total indel frequencies generated in hMSCs (biological replicates: n = 3) with HBB-targeting RNP consisting of either the wildtype Cas9 (WT) or its high-fidelity variant (HiFi). Error bars represent standard deviation. Two-tailed p-value from paired t-test is shown. **f** Off-target activities at a known dominant site for the *HBB*-targeting sgRNA (OT1) as a result of targeting with All-RNA and RNP nucleases are shown. Dots represent frequencies in each biological replicate (All-RNA: n = 10; WT RNP: n = 11; HiFi RNP: n = 3). Bars and error bars represent average frequencies and standard deviation. Two-tailed p-values from paired t-test are shown (Graphpad prism does not show exact p-values < 0.0001).

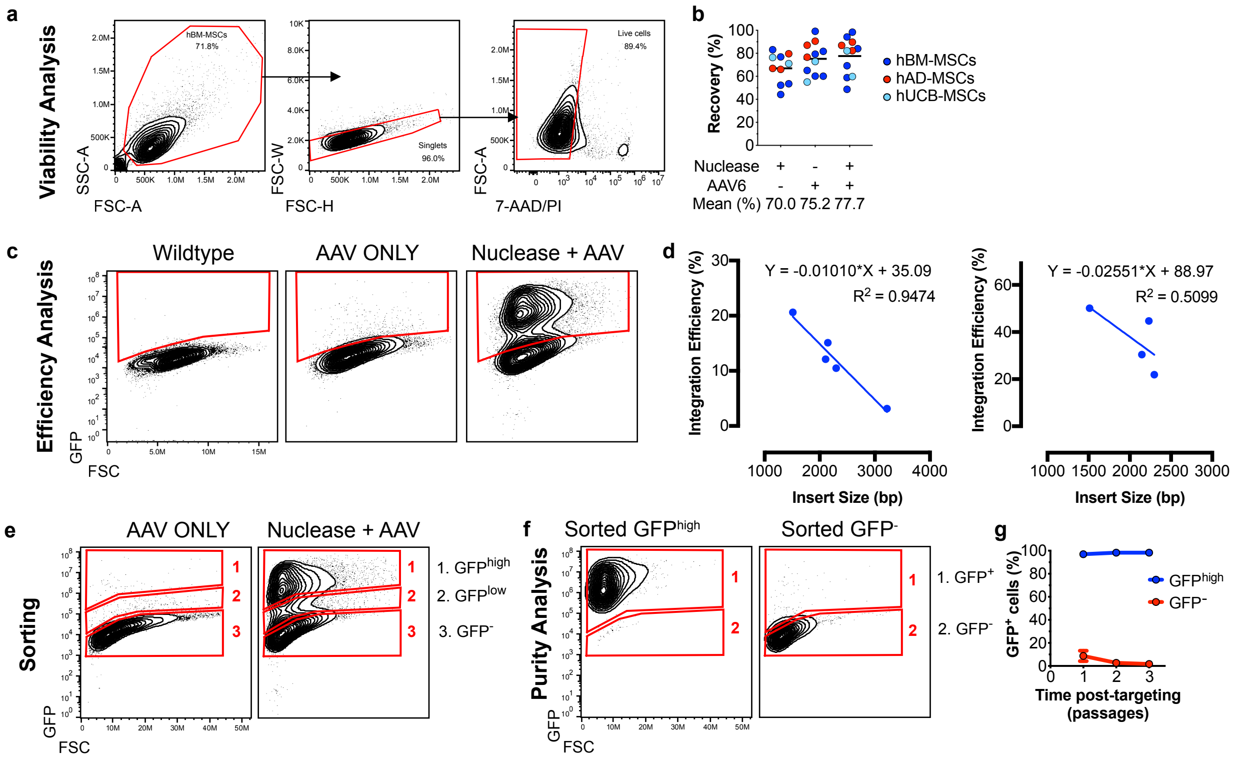

Supplementary Figure 3 | Flow Cytometry analysis and FACS sorting reveal cell viability, efficiency, and purity of Cas9/AAV6-engineered cells

a Human MSCs were identified in FACS contour plots by their forward/side scatter values and single cells were determined by the height and width of forward scatter reads. Dead cells were included by 7-AAD or PI staining. This gating strategy was used to identify single live hMSCs for all subsequent flow cytometry-based analyses and hMSC sorting in this manuscript. **b** Targeted hMSCs were trypsinized after a 24-hour recovery period post-targeting and incubated with 7-AAD or PI and analyzed by flow cytometry to determine viability. Plot represents recovery of hMSCs from different tissue sources at 24 hours following Cas9 nuclease electroporation and/or AAV6 EAT. Each dot represents one biological replicate (Nuclease-only: n = 10; AAV6-only: n = 11; Nuclease+AAV6: n = 11). Bars represent mean values. **c** FACS contour plots show a gating strategy to determine targeting efficiency in Figures 1c-d, and to measure vector delivery efficiencies in Supplementary Figures 1a, 1c-d, 3d, 4, and 6. **d** Scatter plots show linear correlation between insert size and integration efficiency at the *HBB* locus of hMSCs derived from two different human donors. **e** FACS plots show a sorting strategy for hMSC samples targeted with Cas9/AAV system to integrate the SFFV-GFP overexpression cassette and analyzed in Figure 2b-e. **f** FACS plots show a gating strategy for purity analysis of sorted hMSC populations (shown in panel **g**). **g** The GFP^{high} and GFP^{-} (from panel **e**) were sorted, cultured for three additional passages, and purity was analyzed (according to panel **f**). Dots and lines represent changes in purity of GFP-expressing cells in the sorted GFP^{high} and GFP^{-} population (biological replicates: n = 6). Error bars represent standard error of mean.

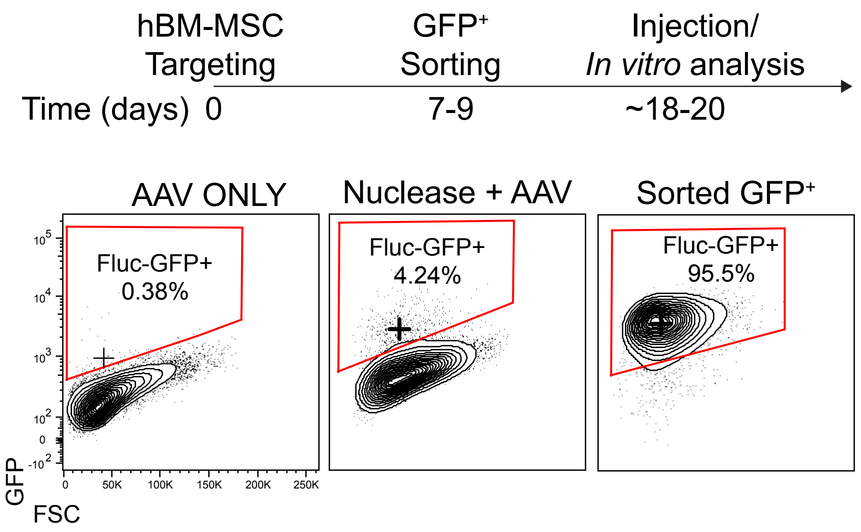

Supplementary Figure 4 | Generation of Fluc MSCs with the Cas9/AAV6 platform.
Using *HBB*-targeting sgRNA, Cas9 mRNA, and Fluc-GFP AAV6 donor, we targeted and purified Fluc$^+$GFP$^+$ hBM-MSCs (abbreviation: Fluc MSCs) for *in vivo* biodistribution and kinetics study (Results shown in Figure 3 and Supplementary Figure 5). FACS plots show distribution and frequency of GFP$^+$ hBM-MSCs in the AAV-only control, Cas9/AAV-targeted, and sorted samples. We achieved 4.24% targeting frequencies in hBM-MSCs and purified the GFP$^+$ population by FACS, which yielded 95.5% GFP$^+$ cells after an additional passage in culture.

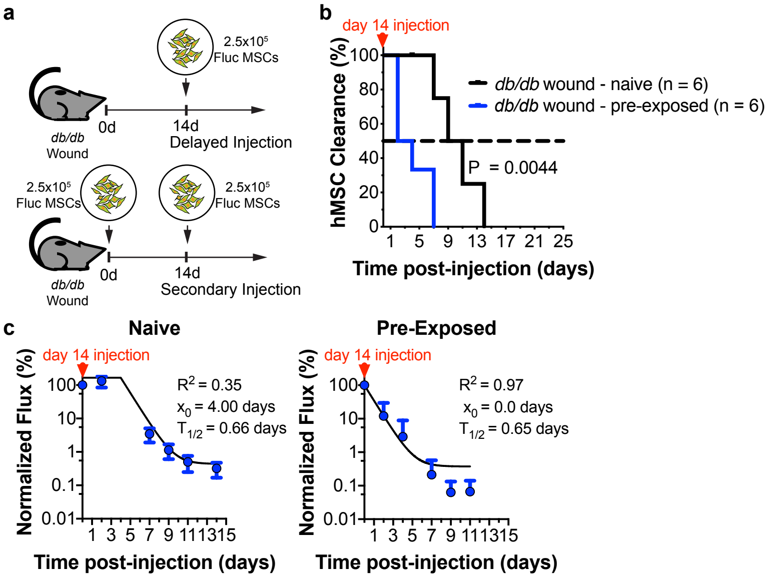

Supplementary Figure S5 | Kinetics of luciferase activities suggested immunogenicity in repeatedly administered hBM-MSCs

a Wounded *db/db* mice were separated into two recipient groups (wounds: n = 6 per recipient group). The 'naïve' group received a single injection of 2.5x10^5 Fluc MSCs at 14 days post-wounding while the 'pre-exposed' group were sensitized with 2.5x10^5 Fluc MSCs injected on the day of wounding and re-administered with the same dose of cells at 14 days post-wounding. Kinetics of luciferase activity were quantified following the day 14 injection. **b** Kaplan-Meyer's plot shows total time to disappearance of luciferase activity from individual wounds in the naïve and pre-exposed recipient groups. Chi-square p-value indicates significant difference of survival curves based on the Log-rank test. **c** Plot shows kinetics of luciferase activity of Fluc MSCs injected at 14 days post-wounding in mice without (left panel, naïve) or with (right panel, pre-exposed) prior exposure to hMSCs. Dots and error bars show relative flux compared to day of injection and standard error of mean. Line shows non-linear curve fit of relative flux as a plateau and exponential decay function. Goodness of fit (R^2), plateau duration (x_0), and half-life ($T_{1/2}$) are shown.

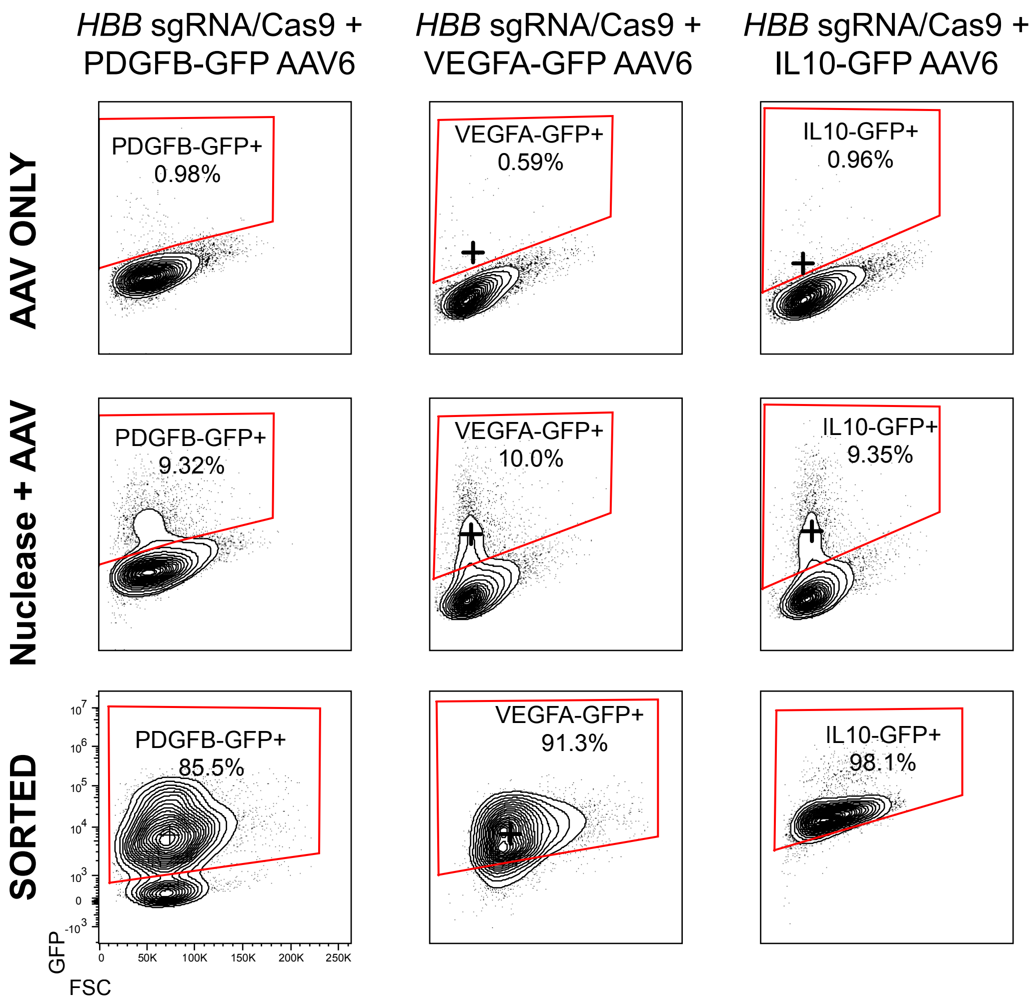

Supplementary Figure 6 | Generation of therapeutic protein-secreting GFP⁺ hBM-MSCs with the Cas9/AAV6 platform

Using *HBB*-targeting sgRNA, Cas9 mRNA, and Therapeutic Factor-GFP AAV6 donors, we targeted and purified Therapeutic Factor^{+}GFP^{+} hBM-MSCs (abbreviation: PDGFB MSCs, VEGFA MSCs, and IL10 MSCs) for *in vivo* efficacy study associated with Figures 4-6 and Supplementary Figures 7-9. As shown in FACS plots, we achieved 10.5% (PDGFB-GFP^{+}), 15.1% (VEGFA-GFP^{+}), and 12.1% (IL10-GFP^{+}) targeting frequencies in hBM-MSCs and purified the GFP^{+} population by FACS, which yielded 85.5%, 91.3%, and 98.1% GFP^{+} cells, respectively, after an additional passage in culture.

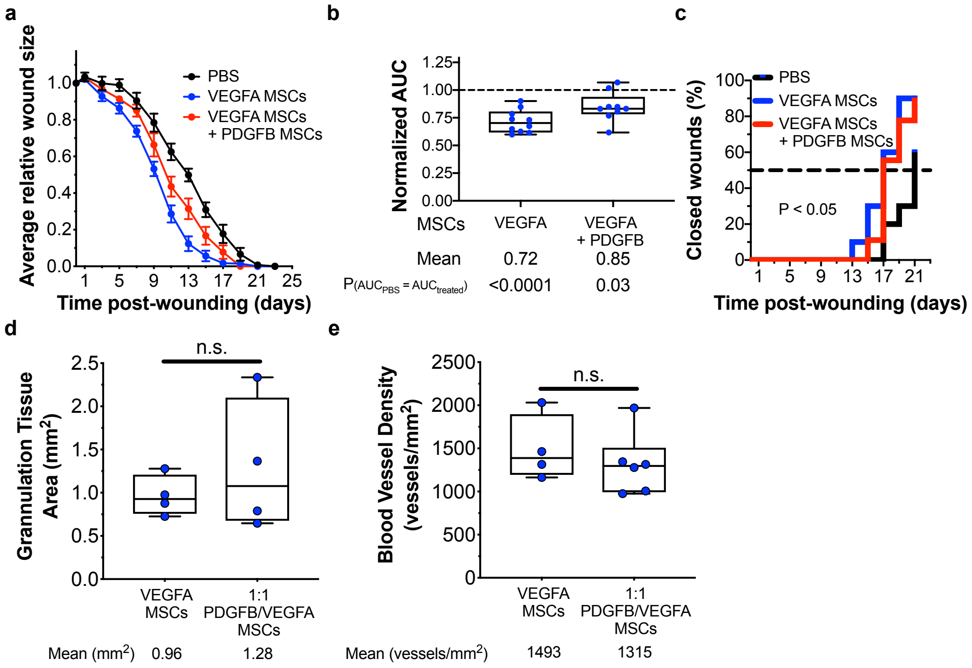

Supplementary Figure 7 | Treatment with 2.5x10⁵ cells with 1:1 ratio of PDGFB MSCs and VEGFA MSCs did not further improve wound healing kinetics from VEGFA MSC-only treatment

a Plot shows changes in average relative wound size (wounds: n = 10 per treatment group) over time in wounds treated with vehicle control (PBS), VEGFA MSCs only, and 1:1 ratio of PDGFB MSCs and VEGFA MSCs at a dose of 2.5×10^5 cells/wound. Dots and lines represent average relative wound size in each treatment group over time. Error bars represent standard error of mean. **b** Box and whiskers plot shows distribution of AUCs for each treatment group (n = 10 wounds per group) when normalized to AUCs of PBS-treated wounds. Individual AUCs are shown. Minima and maxima are bounded by whiskers, lower bounds, centers, and upper bound of boxes represent the 25th, 50th, and 75th percentiles, respectively. P-values shown indicate significant difference of treated wound AUCs and vehicle controls according to One-way ANOVA and Tukey's multiple comparisons test (Graphpad Prism does not show exact p-values below 0.0001). **c** Kaplan-Meyer's plots represent time to complete wound closure of different treatment groups. Chi-square p-values show significant difference in time to closure functions between both VEGFA MSC (p = 0.0169) and 1:1 mixture treated (p = 0.0488) groups over vehicle controls according to Log-Rank test. **d** – **e** Box and whiskers plots show distribution of granulation tissue area (panel **d**, n = 4 wounds per group) and blood vessel density (panel **e**, VEGFA MSCs: n = 4 wounds, 1:1 PDGFB/VEGFA MSCs: n = 6 wounds) of the VEGFA MSC and 1:1 mixture treated groups. Minima and maxima are bounded by whiskers, lower bounds, centers, and upper bound of boxes represent the 25th, 50th, and 75th percentiles, respectively. n.s.: no significant differences ($p < 0.05$) between outcomes of treatment groups shown according to two-tailed, unpaired t-test.

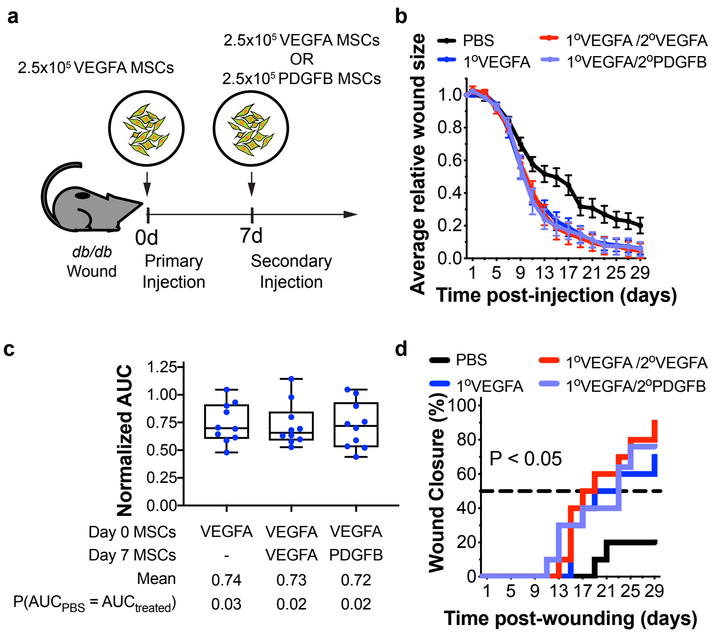

Supplementary Figure 8 | Repeated treatment at day 7 with PDGFB MSCs or VEGFA MSCs did not improve healing kinetics further from initial VEGFA MSC injection

a Wounded *db/db* mice were treated with VEGFA MSCs on the day of wounding and separated into three different treatment groups in which wounds received no further treatment (1°VEGFA), or re-administration with $2.5x10^5$ VEGFA MSCs (1°VEGFA/2°VEGFA) or with $2.5x10^5$ PDGFB MSCs (1°VEGFA/2°PDGFB) at 7 days post-wounding. **b** Plot shows changes in average relative wound size (wounds: n = 10 per treatment group) over time in vehicle control (PBS), and single or serially treated wounds. Dots and lines represent average relative wound size in each treatment group over time. Error bars represent standard error of mean. **c** Box and whiskers plots show distribution of areas under wound curve (AUCs) for each treatment group (n = 10 wounds per group) when normalized to AUCs of PBS-treated wounds. Individual AUCs are shown. Minima and maxima are bounded by whiskers, lower bounds, centers, and upper bound of boxes represent the 25th, 50th, and 75th percentiles, respectively. P-values shown (p = 0.03, 0.02, 0.02) indicate significant difference of treated wound AUCs and vehicle controls according to one-way ANOVA with Tukey's multiple comparisons test. **d** Kaplan-Meyer's plots represent time to complete wound closure of different treatment groups. Chi-square p-values represent significant difference in time to closure functions between all engineered hBM-MSC treated groups (1°VEGFA: p = 0.0223; 1°VEGFA/2°VEGFA: p = 0.0013; 1°VEGFA/2°PDG-FB: p = 0.0190) over vehicle controls according to Log-rank test.

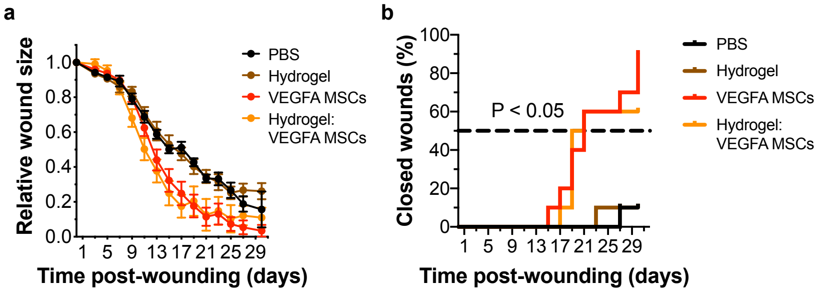

Supplementary Figure 9 | Directly-injected and hydrogel-embedded VEGFA-MSCs yielded similar wound healing kinetics

a Plot shows changes in average wound size over time of both directly injected and embedded VEGFA MSC treatment, as well as vehicle controls (biological replicate: n = 10 wounds per treatment group). Dots and lines represent average relative wound size in each treatment group over time. Error bars represent standard error of mean. **b** Kaplan-Meyer's plot represent time to complete wound closure of different treatment groups. Chi-square p-values (VEGFA MSCs: p = 0.0016; Hydrogel:VEGFA MSCs: p = 0.0150) represent significant difference in time to closure functions between both VEGFA MSC treated groups over PBS vehicle controls according to Log-Rank test.
