# Supplementary material for: Cas9-AAV6-engineered human mesenchymal stromal cells improved cutaneous wound healing in diabetic mice
Source: Nat Commun. 2020 May 18;11:2470. doi: 10.1038/s41467-020-16065-3 (PMC7235221; doi:10.1038/s41467-020-16065-3)
Supplement: Supplementary file 2 — Reporting Summary [file 41467_2020_16065_MOESM2_ESM.pdf]

nature research

Corresponding author(s): Matthew Porteus

Last updated by author(s): 2/17/2020

# Reporting Summary

Nature Research wishes to improve the reproducibility of the work that we publish. This form provides structure for consistency and transparency in reporting. For further information on Nature Research policies, see Authors & Referees and the Editorial Policy Checklist .

## Statistics

For all statistical analyses, confirm that the following items are present in the figure legend, table legend, main text, or Methods section.

| n/a | Confirmed | |
|---|---|---|
| ☐ | ☒ | The exact sample size (*n*) for each experimental group/condition, given as a discrete number and unit of measurement |
| ☐ | ☒ | A statement on whether measurements were taken from distinct samples or whether the same sample was measured repeatedly |
| ☐ | ☒ | The statistical test(s) used AND whether they are one- or two-sided<br>*Only common tests should be described solely by name; describe more complex techniques in the Methods section.* |
| ☐ | ☒ | A description of all covariates tested |
| ☐ | ☒ | A description of any assumptions or corrections, such as tests of normality and adjustment for multiple comparisons |
| ☐ | ☒ | A full description of the statistical parameters including central tendency (e.g. means) or other basic estimates (e.g. regression coefficient) AND variation (e.g. standard deviation) or associated estimates of uncertainty (e.g. confidence intervals) |
| ☐ | ☒ | For null hypothesis testing, the test statistic (e.g. *F*, *t*, *r*) with confidence intervals, effect sizes, degrees of freedom and *P* value noted<br>*Give P values as exact values whenever suitable.* |
| ☒ | ☐ | For Bayesian analysis, information on the choice of priors and Markov chain Monte Carlo settings |
| ☒ | ☐ | For hierarchical and complex designs, identification of the appropriate level for tests and full reporting of outcomes |
| ☒ | ☐ | Estimates of effect sizes (e.g. Cohen's *d*, Pearson's *r*), indicating how they were calculated |

*Our web collection on statistics for biologists contains articles on many of the points above.*

## Software and code

Policy information about availability of computer code

| | |
|---|---|
| Data collection | Images and data were obtained using microscopes, spectral readers, fluorescence/absorbtion/luminescence detection, and flow cytometry with manufacturer provided softwares including Keyence and Leica Imaging softwares, BioTek Gen5, Living Image and Aura bioluminescence imaging softwares, FACS Diva, Cytoflex, and Accuri C6 cytometer softwares. |
| Data analysis | Flow cytometry data was analyzed using Flowjo (v10). IVIS data was analyzed using the Living Image (v 4.7.3) or Aura (v 2.3.1) softwares. Wound size measurement and granulation tissue areas were done with Adobe Photoshop CS6. Wound neovascularization was analyzed using FIJI (ImageJ v 1.52r) with publicly available analysis tools. ELISA spectral absorbance results were analyzed using the Prism8 software. All data plots and curve fit were done on the Prism8 software. |

For manuscripts utilizing custom algorithms or software that are central to the research but not yet described in published literature, software must be made available to editors/reviewers. We strongly encourage code deposition in a community repository (e.g. GitHub). See the Nature Research guidelines for submitting code & software for further information.

## Data

Policy information about availability of data

All manuscripts must include a data availability statement. This statement should provide the following information, where applicable:

- Accession codes, unique identifiers, or web links for publicly available datasets
- A list of figures that have associated raw data
- A description of any restrictions on data availability

All data that supports conclusion stated are included in the manuscript.

# Field-specific reporting

Please select the one below that is the best fit for your research. If you are not sure, read the appropriate sections before making your selection.

☒ Life sciences ☐ Behavioural & social sciences ☐ Ecological, evolutionary & environmental sciences

For a reference copy of the document with all sections, see nature.com/documents/nr-reporting-summary-flat.pdf

# Life sciences study design

All studies must disclose on these points even when the disclosure is negative.

| | |
|---|---|
| Sample size | Sample sizes were chosen based on cell and animal availability or number of experimental or control groups needed to draw conclusions, but were not calculated prior to experiments. The resulting data were sufficient to show significance of reported data based on magnitudes of differences between groups. |
| Data exclusions | Data were excluded based on ROUT Outlier Test (FDR: Q = 1%), or based on failed experiments that include failed reagent preparations. |
| Replication | All attempts at replication were successful. Reproducibility of biochemical assays were confirmed by technical replicates, while reproducibility of gene targeting methods and wound healing efficacies were confirmed by biological replicates. Cas9/AAV6 targeting of hMSCs from 12 human donors were performed collectively in 10 total independent experiments using 2-3 technical replicates to measure efficiencies. All wound healing data were obtained collectively from 5 independent animal studies, using 10 wounds per treatment group. Bioluminescence imaging data were obtained collectively from 3 independent experiments. |
| Randomization | Randomization of cell donors were indirect since cells were obtained based on availability from multiple commercial and academic sources. Mice were assigned treatment groups randomly based on order of mice receiving injection. |
| Blinding | For all ex vivo experiments, investigators were not blinded to experiment groups at the time of data collection for cells analyzed by microscopy, flow cytometry, differentiation assays and molecular analyses. The lead author performed experimental steps, assigned treatment groups, and analyzed all data on their own while occasionally verifying transgene expression in-culture and harvest cells for further analyses on cells ex vivo with treatment group identification written on culture vessels. Therefore, the author was aware of the treatment groups and their corresponding culture vessels during the experiment. However, all ex vivo experiments contained appropriate negative controls. For in vivo experiments, investigators were blinded at the time wound photos were taken at all time points since animals are assigned tag numbers without treatment group identification on the animals or their cages. Investigators were blinded to group assignment at the time of wound data analyses. |

# Reporting for specific materials, systems and methods

We require information from authors about some types of materials, experimental systems and methods used in many studies. Here, indicate whether each material, system or method listed is relevant to your study. If you are not sure if a list item applies to your research, read the appropriate section before selecting a response.

## Materials & experimental systems

| n/a | Involved in the study | |
|---|---|---|
| ☐ | ☒ | Antibodies |
| ☒ | ☐ | Eukaryotic cell lines |
| ☒ | ☐ | Palaeontology |
| ☐ | ☒ | Animals and other organisms |
| ☒ | ☐ | Human research participants |
| ☒ | ☐ | Clinical data |

## Methods

| n/a | Involved in the study | |
|---|---|---|
| ☒ | ☐ | ChIP-seq |
| ☐ | ☒ | Flow cytometry |
| ☒ | ☐ | MRI-based neuroimaging |

## Antibodies

| | |
|---|---|
| Antibodies used | Antibodies used for flow cytometry were obtained from BioLegend Inc, with clone numbers stated in the methods section. Immonohistochemistry staining used antibodies provided by Histowiz Inc. Antibodies obtained from BioLegend Inc. (San Diego, CA) included the following (Cat no.-Description-(Clone)-lot no.):<br>323217 CD105-PE/Cy7 (Clone 43A3) lot no. B221405,<br>344005 CD73-APC (Clone AD2) lot no. B252652,<br>328117: CD90-PerCP/Cy5.5 (Clone 5E10) lot no. B253074,<br>301816: CD14-Pacific Blue (Clone M5E2) lot no. B257027,<br>363035: CD19-Pacific Blue (Clone SJ25C1) lot no. B226989,<br>343511: CD34-Pacific Blue (Clone 581) lot no. B234481,<br>304021: CD45-Pacific Blue (Clone 2D1) lot no. B253656, and,<br>307623: HLA-DR-Pacific Blue (Clone L243) lot no. B254803. |

| | |
|---|---|
| Validation | BioLegend Inc validated antibodies used for flow cytometry.<br>Histowiz Inc validated antibodies used for immunohistochemistry and provided staining of positive controls.<br><br>All antibodies used here have been previously reported and are routinely used in flow cytometry studies. Per vendors' websites, they take quality control measures to ensure that all antibodies sold are valid and reproducible. All antibodies from Biolegend listed here are also listed in the Antibody Registry, which is part of the Resource Indentification Initiative (https://scicrunch.org/resources).<br><br>For flow cytometry, positive staining were validated using positive (antigen positive cells) and negative (isotype) controls with the recommended antibody concentrations from the manufacturers. |

## Animals and other organisms

Policy information about studies involving animals; ARRIVE guidelines recommended for reporting animal research

| | |
|---|---|
| Laboratory animals | 13-14 week old female db/db mice and 6-8 week old NSG mice (male and female) were used for in vivo studies |
| Wild animals | Wild animals were not used. |
| Field-collected samples | Study did not involve field data collection. |
| Ethics oversight | Stanford University approves APB and APLAC protocols used in this study. |

Note that full information on the approval of the study protocol must also be provided in the manuscript.

## Flow Cytometry

### Plots

Confirm that:

- [x] The axis labels state the marker and fluorochrome used (e.g. CD4-FITC).
- [x] The axis scales are clearly visible. Include numbers along axes only for bottom left plot of group (a 'group' is an analysis of identical markers).
- [x] All plots are contour plots with outliers or pseudocolor plots.
- [x] A numerical value for number of cells or percentage (with statistics) is provided.

### Methodology

| | |
|---|---|
| Sample preparation | Human mesenchymal stromal cells used in flow cytometry were obtained from adherent monolayer cultures by trypsinization without tissue processing steps. If needed, antibody staining was done using standard protocols and with isotype controls. |
| Instrument | Accuri c6, BD FACS Aria II, and Beckman Coulter Cytoflex instruments were used to collect cytometry data. |
| Software | Accuri c6, BD FACS Diva, and CytExpert software were used to collect data. All data are analyzed using Flowjo v10. |
| Cell population abundance | Abundance of cell population post-sort were stated in the manuscript when appropriate. Histograms were staggered to show differences in MFI when appropriate, therefore, not all FACS plot are contour plots. |
| Gating strategy | Gating for whole populations was done by FSC/SSC scatter, single cell scatter gating, and dead cell exclusion. Positive signal gating was done against negative controls and is provided when necessary in the supplementary information. |

- [x] Tick this box to confirm that a figure exemplifying the gating strategy is provided in the Supplementary Information.
